# Groundwater Microbial Diversity Associated With Icelandic Basaltic Subsurface Environments

**DOI:** 10.1111/1758-2229.70238

**Published:** 2025-11-30

**Authors:** Juliette Bas‐Lorillot, Bénédicte Ménez, Bastien Wild, Guillaume Borrel, Manon Le Bihan, Andri Stefánsson, Jóhann Gunnarsson‐Robin, Anna Bríet Bjarkadóttir, Sigríður María Aðalsteinsdóttir, Delphine Tisserand, Damien Daval, Emmanuelle Gérard

**Affiliations:** ^1^ Institut de Physique du Globe de Paris, Université Paris Cité, CNRS Paris France; ^2^ ISTerre, CNRS, IRD, Université Gustave Eiffel, Université Grenoble Alpes, Université Savoie Mont Blanc Grenoble France; ^3^ Evolutionary Biology of the Microbial Cell Institut Pasteur, Université Paris Cité Paris France; ^4^ Institute of Earth Sciences, University of Iceland Reykjavík Iceland

**Keywords:** basalts, deep ecosystems, geothermal groundwater, silicate dissolution, subsurface alteration

## Abstract

Microbial communities in the deep basaltic aquifers of Iceland remain poorly characterized, despite their relevance for understanding subsurface biogeochemical processes, including silicate weathering. Here, we used 16S rRNA gene metabarcoding to investigate bacterial and archaeal diversity in 22 geothermal wells spanning broad gradients in temperature (30°C–110°C), pH (7.0–11.0), and bedrock age (0.01–15 Myr). We observed highly variable microbial assemblages, with several dominant taxa affiliated with known deep biosphere lineages, including hydrogenotrophs and sulfate reducers. Archaeal communities were less diverse and displayed domain‐specific patterns, distinct from bacterial assemblages. Beta diversity was primarily structured by temperature and pH, and, to a lesser extent, by bedrock age. Thermodynamic and kinetic parameters derived from groundwater chemistry—including redox potential and silicate dissolution rates—also accounted for significant fractions of the variation in microbial beta diversity, although it cannot be ruled out that their influence primarily reflected underlying correlations with temperature and pH. Our results suggest that both environmental gradients and host‐rock reactivity shape microbial diversity in these systems. This highlights the importance of considering geochemical context when designing subsurface microcosm experiments, and identifies candidate taxa for future studies exploring links between microbial composition and silicate weathering processes.

## Graphical abstract

Overview of microbial diversity and environmental drivers in Icelandic deep basaltic aquifers. Using 16S rRNA gene metabarcoding across 22 geothermal wells spanning broad temperature, pH, and bedrock‐age gradients, the study reveals distinct bacterial and archaeal community structures shaped by temperature, pH, and host‐rock reactivity. Findings highlight key ecological and geochemical controls relevant to subsurface silicate weathering and microcosm experiment design.

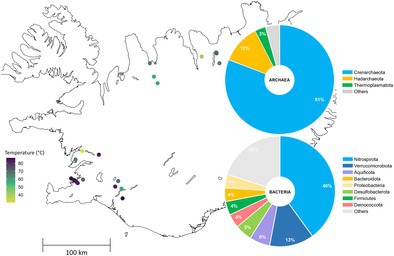

**Abbreviations**
ANOVA: analysis of variance
ASVs: amplicon sequence variants
CCSU: carbon capture, storage and utilisation
DOC: non‐purgeable dissolved organic carbon
HAC: hierarchical agglomerative clustering
PCoA: principal coordinates analysis
PCR: polymerase chain reaction
PERMANOVA: permutational multivariate analysis of variance
SI: saturation index
TN: total nitrogen
TST: Transition State Theory

## 1 Introduction

Silicate weathering is a cornerstone of Earth's geochemical cycles, regulating the long‐term carbon balance of the atmosphere through carbon dioxide (${\mathrm{CO}}_{2}$) consumption, while also contributing to soil genesis and supplying ecosystems with essential nutrients (Berner et al. 1983; Gaillardet et al. 1999) and energy sources (e.g., Napieralski et al. 2019). In addition, the dissolution of silicates is the rate‐limiting step in mineral carbonation, one of the carbon capture, storage and utilisation (CCSU) technologies, which aim to reduce ${\mathrm{CO}}_{2}$ emissions and mitigate climate change (e.g., Daval 2018). While silicate weathering has long been described as dominantly abiotic—controlled by physicochemical parameters such as temperature, pH, fluid saturation state, as well as mineral composition and structure of the host rock or soil—growing evidence shows that microorganisms can play a critical role in accelerating and directing silicate weathering under a wide range of environmental conditions (e.g., Bennett et al. 1996; Rogers and Bennett 2004; Uroz et al. 2009; Gadd 2010).

Microorganisms alter silicate surfaces through a combination of direct and indirect mechanisms, including excretion of organic acids and siderophores, redox transformations of structural metals, physical penetration, and biofilm‐mediated microenvironmental modifications (e.g., Banfield et al. 1999; Lian et al. 2008; Kruber et al. 2008; Cockell et al. 2009; Gadd 2010; Sudek et al. 2017; Wild et al. 2021). These processes have been most extensively documented in near‐surface environments such as soils and rhizosphere systems, where microbial communities are readily accessible for in situ experiments (e.g., Wild et al. 2018, 2019), as well as sampling and characterisation, including through cultivation‐based approaches that allow further use of isolated species in experiments (e.g., Shirokova et al. 2012; Stockmann et al. 2012; Stranghoener et al. 2018). Despite this, laboratory studies investigating microbially driven mineral dissolution mostly rely on model organisms chosen for their convenience and tractability rather than for their environmental representativeness. This could have led to ecological mismatches between the microorganisms used in experiments and those that naturally inhabit mineral interfaces, especially in more extreme or inaccessible environments. Indeed, the majority of Earth's microbial biomass does not reside at the surface, but in the terrestrial and marine subsurface (Whitman et al. 1998; Stevens and McKinley 1995; Bar‐On et al. 2018; Magnabosco et al. 2018, 2019; Flemming and Wuertz 2019; Templeton and Caro 2023). Despite its vast extent and importance in global biogeochemical cycling and therefore likely silicate weathering, the deep biosphere remains an environment poorly explored in terms of microbial diversity, metabolic capacity, and ecological function (Momper et al. 2023; Soares et al. 2023; Beaver and Neufeld 2024). This still limited exploration is largely due to the complex sampling procedures required (Santelli et al. 2010; Morono 2023) and the difficulty of ensuring representative samples, given the low biomass characterising subsurface environments—from $10^{2}$ to $10^{7}$ cells mL^−1^ in fracture water, and from $10^{1}$ to $10^{5}$ cells g^−1^ in low porosity crustal rocks with typically $10^{3}$ to $10^{5}$ cells mL^−1^ in basalt‐hosted fluids (Templeton and Caro 2023)—that increases the risk of contamination. Despite sometimes imperfect sampling and the fact that some efforts were opportunistic rather than specifically dedicated to microbial ecology, significant progress has been made over the past few decades. This progress has shown, in particular, that deep ecosystems have their own specific characteristics and composition, which depend notably on the host rock and its architecture (Osburn et al. 2014; Bochet et al. 2020; Casar et al. 2020, 2021; Colman et al. 2025). These features make it difficult to extrapolate findings from surface‐based experiments to deeper crystalline systems with variable lithologies. Moreover, a significant portion of the deep biosphere comprises species that remain unknown, and elucidating their nature and distribution remains a key area of ongoing research (Beaver and Neufeld 2024).

By way of consequence, although advanced experimental setups now enable detailed monitoring of abiotic and biologically‐mediated mineral dissolution and rate estimates (Wild et al. 2022), assessing the role of subsurface microorganisms in silicate alteration remains an open and challenging question. The extremely limited number of cultivated strains from the deep biosphere (Lloyd et al. 2018) risks overlooking key biological strategies that exist in the subsurface—such as (slow‐growing) chemolithotrophy under sometimes reduced redox potential, extreme oligotrophy, as well as temperature and pH‐dependent metabolisms (e.g., Orsi 2018; Trembath‐Reichert et al. 2019)—and their potential impact on mineral alteration. Alternatively, micro‐ and mesocosms can be used (e.g., Napieralski et al. 2019; Sengupta et al. 2019; Chikkanna et al. 2021), but these must be ecologically representative of the wide range of environmental conditions found in the subsurface where host‐rock composition and availability of dissolved compounds in groundwater also influence which organisms and metabolisms are supported (e.g., Simkus et al. 2016; Magnabosco et al. 2018). This is particularly the case in basaltic environments such as mid‐ocean ridges or volcanic islands where temperature, salinity, pH, and redox conditions greatly vary depending on fluid origin, residence time, depth of circulation, and the extent of water–rock interactions and magmatic degassing. Basalts, which are now acknowledged to represent one of the largest, if not the largest, microbial habitats on Earth (Cowen et al. 2003; Edwards et al. 2005; Heberling et al. 2010), are also among the most seriously considered targets for ${\mathrm{CO}}_{2}$ mineral storage in CCSU operations, as the ones deployed in Iceland thanks to the long‐standing CarbFix project (Matter et al. 2011). However, our ecological understanding of microbial life at depth in these settings remains limited, making it challenging to assess the microbial contribution to subsurface silicate weathering, predict microbial responses to ${\mathrm{CO}}_{2}$ injection (e.g., Trias et al. 2017), and ultimately, design ecologically representative microcosms for mineral dissolution experiments.

Here we leveraged the geothermal infrastructure in Iceland, where numerous wells provide access to basalt‐hosted groundwaters spanning a wide range of temperature, pH, and salinity conditions, thereby enabling a deeper characterisation of microbial ecology within subsurface environments. By integrating geochemical, thermodynamic and microbial community analyses, we aim to define a set of representative environmental end‐members that can serve as the foundation for building ecologically‐informed laboratory microcosms. Identifying the microbial players involved and the constraints they face is essential for assessing their role in subsurface weathering and for designing efficient biogeochemical models of ${\mathrm{CO}}_{2}$ sequestration and mineral transformation in the subsurface.

## 2 Methods

### 2.1 Groundwater Collection and Analyses

#### 2.1.1 Sampling Procedure

**FIGURE 1:**
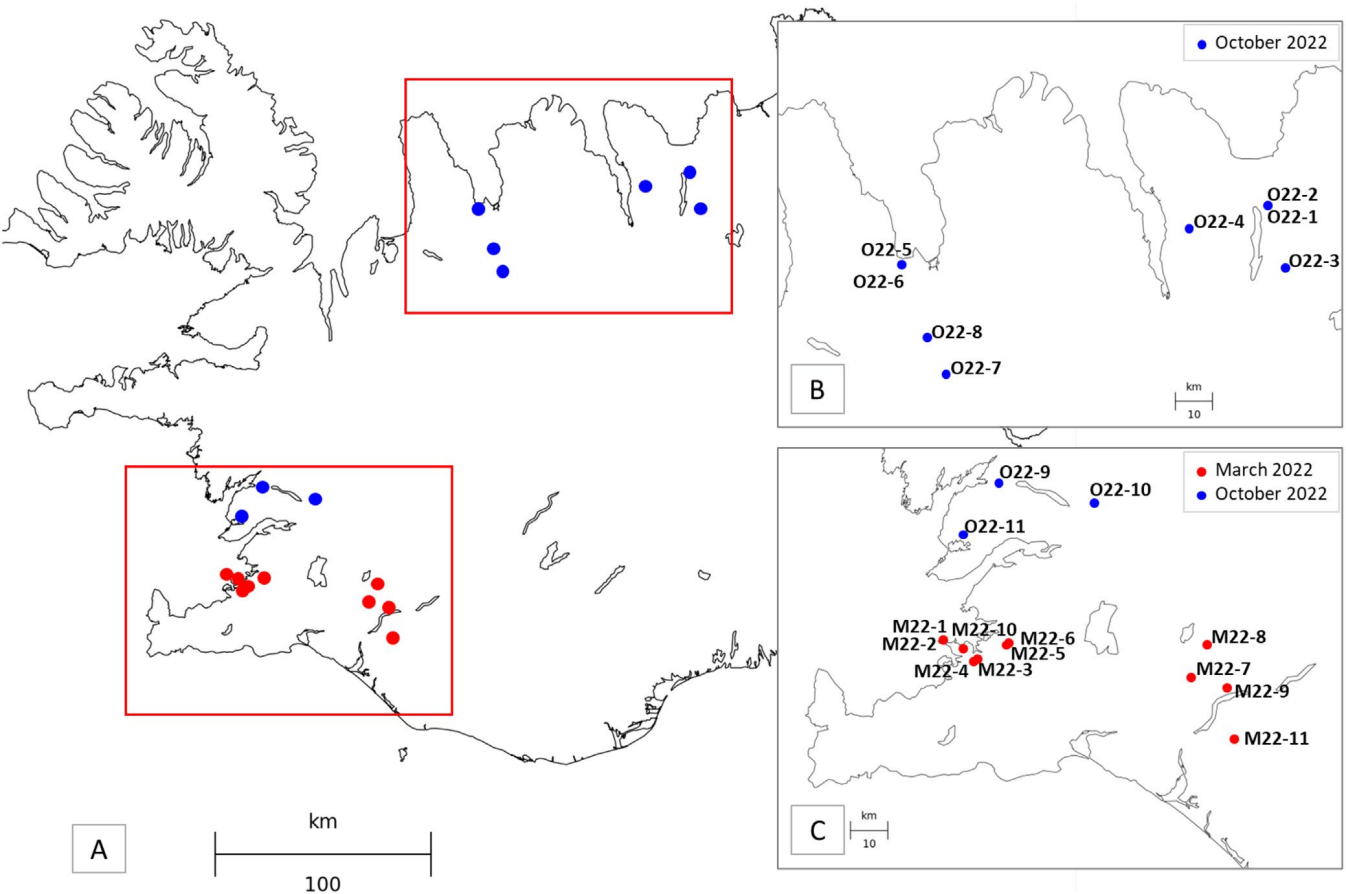
(A) Locations of the Icelandic geothermal wells sampled during March (red dots) and October (blue dots) 2022 field campaigns. Insets (B) and (C), where well names are indicated, provide enlarged views of the Skagafjörður area and the Reykjanes peninsula in the lowlands, respectively. Coordinates of all sampled wells are given in Table S1.

Field campaigns were conducted in Iceland in March and October 2022 to collect groundwater samples from 22 wells tapping into basaltic subsurface environments. The wells were distributed across the southwestern and northern regions of Iceland (Figure 1 and Table S1) and were selected for the contrasted physicochemical properties of their geothermal waters. In order to sample groundwater under a wide range of environmental conditions, all available opportunities for well access were utilized. As a result, the wells varied considerably in terms of infrastructure, from cased and instrumented wells operated by geothermal energy companies to basic farm wells on private land. Given the uneven characterization of the bedrock mineralogy across sites, the primary geological parameter used to differentiate between wells was the age of the bedrock (Table S1), which was initially retrieved from Jóhannesson (2014). These ages were subsequently refined by reviewing well‐specific reports deposited in the database operated by the Icelandic National Energy Authority (Orkustofnun; https://www.map.is/os/), when available. Since the depth of the wells targeted in the present study ranged from a few tens of meters to several kilometers (Table S1), some intersect multiple geological units whose age differs markedly from that indicated on the geological map. However, except for four wells (i.e., O22‐1, O22‐2, O22‐5, and O22‐6), the bedrock age indicated by Jóhannesson (2014) was deemed reliable for characterizing all other wells.

Each well was sampled for both chemical and DNA analyses. The sampling system, connected to the wellhead, comprised sterile flexible silicone tubing, rigid high‐density polyethylene tubing, and a stainless‐steel cooling coil (Arnórsson et al. 2006). Groundwater temperature at the outlet of the cooling circuit was maintained below 40°C to preserve the filter integrity. Prior to sample collection, the system was systematically flushed with groundwater from the respective well for approximately 30 min. This flushing step aimed to purge stagnant water when necessary, remove the largest corrosion and/or rock particles and microbial biofilms from the well and tubing walls, and ensure the collection of groundwater that was as representative as possible of in situ conditions (see also Section 4.3).

#### 2.1.2 Chemical Analyses

Temperature, pH, and conductivity were measured on site, using a WTW Multi 340i handheld multimeter calibrated before use. Typical uncertainties were ±2°C for temperature, ±0.1 unit for pH, and ±10% for conductivity. Dissolved dioxygen ($O_{2\left(\mathrm{aq}\right)}$) concentrations were also measured in the field using an optical oxygen sensor (NeoFox Sport Phase fluorometer, Ocean Optics).

Dissolved hydrogen sulphide (H_2_S) and ${\mathrm{CO}}_{2}$ concentrations were quantified either by titration of steam condensate, or following redissolution in alkaline solution within evacuated gas sampling bulbs, in accordance with the procedures described by Arnórsson et al. (2006).

Groundwater samples were filtered on site using 0.22 μm cellulose acetate filters. Filtrates were collected and stored in polypropylene tubes for laboratory cation and anion analyses. Samples for cation analyses were acidified with concentrated HNO_3_ (Merck Suprapur, 1% vol.) while samples for anion analysis were left untreated. Major cations were analysed by inductively coupled plasma‐optical emission spectrometry using a Thermo Scientific iCap system and major anions by ion chromatography using a Thermo Scientific Dionex 2000 system at the Institute of Earth Sciences of the University of Iceland (Reykjavík, Iceland). Major cations and anions were quantified with analytical precisions better than 5% and 3%, respectively, with typical detection limits below 0.01 ppm for cations and anions. Filtered samples for non‐purgeable dissolved organic carbon (DOC) and total nitrogen (TN) analyses were collected and stored in amber glass vials. DOC and TN analyses were conducted using a Shimadzu TOC‐VCSN analyser at the geochemistry‐mineralogy platform of ISTerre (OSUG, Grenoble, France) following the protocols detailed in Tisserand et al. (2022, 2024). The quantification limits were about 0.1 mg L^−1^.

#### 2.1.3 Microbial Biomass Collection

Microbial biomass from groundwater was collected for DNA analysis using established best practices to preserve sample integrity, minimise contamination, and maximise the representativeness of in situ microbial communities (Santelli et al. 2010; Morono 2023), consistent with procedures employed in similar investigations (e.g., Trias et al. 2017; Giroud et al. 2025). Biomass was collected by filtering for each replicate 10–12 L of groundwater through a sterile 0.22 μm Sterivex‐GP filtration unit (Millipore). To prevent clogging and limit contamination—from corrosion products originating from the well casing, as well as microbial biofilms potentially detached and carried by fluid flow—a sterile 5.0 μm prefilter (Millex‐SV filter unit with polyvinylidene fluoride membrane; Millipore) was connected upstream of the Sterivex‐GP filtration unit. Filtration duration varied from several hours to half a day, depending on the flow rate most often imposed by the well pump system. When field conditions allowed, groundwater sampling and filtering were either replicated on site, or groundwater was directly collected from the cooling circuit into sterile low‐density polyethylene bags (Thermo Scientific Labtainer BPC 20 L), prior to overnight filtration using the filter assembly described above.

Duplicate samples were collected for all wells in October 2022, except for wells O22‐1 and O22‐6. Triplicate samples were obtained for well O22‐4. Samples from the March 2022 campaign were not duplicated.

At the end of filtration, Sterivex‐GP filtration units were either filled with sterile absolute ethanol or drained of residual water, sealed with sterile Luer Lock caps and stored in sterile Falcon tubes. They were kept at −20°C during the field campaign and subsequently stored at −80°C until DNA extraction.

#### 2.1.4 DNA Extraction and Sequencing

Microbial DNA was extracted from the Sterivex‐GP filtration units under a laminar flow hood using the DNeasy PowerWater Sterivex kit (Qiagen) following the manufacturer's protocol and then stored at −80°C. All extractions were carried out within 2 weeks of sample collection. To control for contamination, procedural blanks were systematically processed in parallel using identical conditions. These included: (i) a pristine Sterivex‐GP filtration unit opened under the laminar flow hood without exposure to an environmental sample, and (ii) a Sterivex‐GP filtration unit connected to the field tubing system, flushed with heat‐sterilised Milli‐Q water to replicate field handling. DNA concentrations were assessed for each sample using a Qubit 4 fluorometer with a Qubit dsDNA HS Assay kit (Invitrogen) according to the manufacturer's instructions. For each sample, measurements were performed in triplicate and averaged (Table S1).

Illumina MiSeq paired‐end tag sequencing of the 16S rRNA gene was performed at the Marine Biological Laboratory (Woods Hole Oceanographic Institution, MA, USA). Standard barcoded primers for bacteria and archaea were used to amplify, using polymerase chain reaction (PCR), the hypervariable region V4–V5 of the targeted gene (518F 5′CCAGCAGCYGCGGTAAN3′/926R 5′ANTYAAANRAATSGACGG3′ for bacteria and 517F 5′GYYTAAARNRYYYGTAGC3′/958R 5′CCGGCGTTGANTCCAATT3′ for archaea) following the protocol proposed by Nelson et al. (2014). All raw reads were submitted to the NCBI Sequence Read Archive under Bioproject ID PRJNA1347816.

### 2.2 Metabarcoding and Statistical Analyses

Raw sequences were processed using FROGS v4.0.1 (Escudié et al. 2018) on the open source, web‐based Galaxy platform, utilizing the public servers at https://usegalaxy.fr and https://vm‐galaxy‐prod.toulouse.inrae.fr. First, the high‐quality paired‐end reads were merged using FLASH (Magoč and Salzberg 2011) with a mismatch tolerance of 0.1. Sequences lacking primers were discarded using cutadapt (Martin 2011). Next, amplicon sequence variants (ASVs) were inferred using SWARM following the guidelines from version 3.2 with a clustering aggregation distance of 1. Chimeric sequences were identified and removed using VSEARCH with the *de novo* UCHIME algorithm (Edgar et al. 2011; Rognes et al. 2016). Low‐abundance clusters (< 0.005% of total reads) were filtered out.

Microbial diversity analyses were performed in RStudio (R version 4.2.3) using the output BIOM and tree files from FROGS. All visualisations were generated using the ggplot2 R package (Wickham 2016). For downstream analyses, ASVs assigned to archaea were removed from the bacterial dataset and vice versa.

Sequence depth ranged between 18,038 (well M22‐8) and 329,197 (well O22‐9) reads per sample for bacteria, and from 355 (well M22‐5) to 131,922 (well O22‐9) reads for archaea. To standardise sequencing effort, datasets were rarefied using the rarefy_even_depth() function from the phyloseq package in RStudio (McMurdie and Holmes 2013) with a seed value of 500,000. Rarefaction thresholds were set at 18,038 and 6286 reads for bacteria and archaea, respectively (Figure S1). During this process, no ASVs were lost for the bacterial dataset but two ASVs were lost for the archaeal dataset. Due to insufficient sequencing depth and rarefaction curves not reaching a plateau (Figure S1B), archaeal data from wells M22‐5 and M22‐8 were further excluded from downstream community structure analysis. No bacterial data were discarded, as all the rarefaction curves reached a plateau at the selected sequencing depth of 18,038 (Figure S1A).

Microbial community composition was assessed with principal coordinates analysis (PCoA) based on unweighted UniFrac distance matrices computed using the ordinate() function of phyloseq (McMurdie and Holmes 2013) and plotted using vegan (function envfit()) (Oksanen et al. 2019) and ggplot2 (Wickham 2016). Hierarchical agglomerative clustering (HAC) using the Ward method was also applied to unweighted UniFrac distance matrices to assess beta diversity and group samples based on the phylogenetic relationships of the microorganisms present. Dendrograms were visualised using the dendextend R package. Statistical differences in community composition were evaluated with PERMANOVA (999 permutations), as implemented in vegan (function adonis2()). Analyses were conducted separately for each parameter of interest, followed by combinations of non‐correlated parameters. Samples lacking data for a given parameter were excluded from the corresponding analyses.

Alpha diversity was assessed with the Observed ASVs metric from the phyloseq R package. To assess the influence of the main environmental variables on alpha diversity, analysis of variance (ANOVA) was performed using the aov() function from the base R stats package. Note that wells O22‐4 and O22‐11 were excluded from the ANOVA analysis due to incomplete input data.

In addition to the taxonomic assignments—against the SILVA 16S rRNA gene database release 138.1 (Quast et al. 2012)—provided by the FROGS pipeline (Escudié et al. 2018), sequence similarity analyses were performed for the 20 most abundant bacterial and archaeal ASVs using the BLAST+ tool (https://blast.ncbi.nlm.nih.gov/Blast.cgi, Camacho et al. 2009) against the NCBI non‐redundant nucleotide database (Goldfarb et al. 2025).

### 2.3 Thermodynamic and Kinetic Calculations

High‐temperature pH (i.e., pH at the temperature measured at the wellhead, hereafter referred to as pH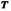), redox potential ($E_{H}$) and Gibbs free energy (ΔG) of olivine and basaltic glass dissolution (i.e., the key Fe‐bearing silicate phases that make up the basaltic subsurface) were calculated using the JCHESS 2.0 software (van der Lee and De Windt 2002), based on groundwater chemical analyses. The chemical equations considered to run these calculations were:

(1) $${\mathrm{Mg}}_{2}{\mathrm{SiO}}_{4}+4H^{+}\to {\mathrm{SiO}}_{2\left(\mathrm{aq}\right)}+2{\mathrm{Mg}}^{2+}+2H_{2}O$$

(2) $${\text{SiAl}}_{0.35}{\left(\mathrm{OH}\right)}_{1.05}+1.05H^{+}\to {\mathrm{SiO}}_{2\left(\mathrm{aq}\right)}+0.35{\mathrm{Al}}^{3+}+1.05H_{2}O$$

for olivine and basaltic glass dissolution, respectively (note that the so‐called ‘hydrated basaltic glass’ composition reported by Galeczka et al. (2014) was used as a proxy for basaltic glass).

To ensure accurate modelling, the electrical balance was adjusted so that the pH calculated at 20°C (pH_20°C_) matched the value measured at ~20°C. This was achieved by varying concentrations within ±20% of their measured analytical values. This adjustment resulted in calculated pH_20°C_ values that were within ±0.2 pH unit of the ones measured at ~20°C, except for wells M22‐5 (ΔpH = 0.3), O22‐1 (ΔpH = 0.5), and O22‐2 (ΔpH = 0.4), with ΔpH corresponding to the difference between the calculated and measured pH values at 20°C. For these wells, concentrations were not further adjusted, as they would exceed by far the analytical uncertainty threshold (≤ 10%). In these cases, poor agreement was attributed either to chemical species that were not measured, despite contributing to the electrical balance, or to a possible pH drift between groundwater sampling and measurement (typically a brief delay of a few minutes to allow the sample to reach approximately 20°C).

Once the pH was adjusted, the saturation indices (*SI*) of the studied phases as provided by the JCHESS code were used to calculate ΔG according to:

(3) $$\Delta G=\mathrm{RT}\times \mathrm{SI}\times \ln\left(10\right)$$

where *R* is the universal gas constant (in J mol^−1^ K^−1^), and *T* the temperature measured at the wellhead (in K). Note that the considered equilibrium constant for basaltic glass is that of the hydrated basaltic glass surface suggested by Galeczka et al. (2014).

To assess the redox potential of the collected groundwater, it was assumed that the ${{\mathrm{SO}}_{4}}^{2-}$/H_2_S redox couple controls the oxygen fugacity of the groundwater, in line with previous studies (e.g., Snæbjörnsdóttir et al. 2017). For each well, the equilibrium constant (log *K*) of the ${{\mathrm{SO}}_{4}}^{2-}$/H_2_S redox couple was calculated using known values of log *K* reported in the literature (Stefánsson et al. 2005). The activities *a*_*i*_ of ${{\mathrm{SO}}_{4}}^{2-}$, H_2_S, and $H^{+}$ species together with log *K* calculated at the measured wellhead temperature were used to calculate $E_{H}$ following:

$$E_{H}=E_{H}^{0}+\frac{\mathrm{RT}}{\mathrm{nF}}\sum_{i}\nu_{i}\ln\left(a_{i}\right)$$

where $E_{H}^{0}$ is the standard redox potential (in V), *n* is the number of electrons transferred, *F* is the Faraday constant (in C mol^−1^), and $\nu_{i}$ are the stoichiometric coefficients for the considered aqueous species. As equilibrium among multiple redox pairs is unlikely in Icelandic geothermal systems (Stefánsson and Arnórsson 2002; Stefánsson et al. 2005), the resulting $E_{H}$ values should only be considered as indicative, and were primarily used to test whether the microbial diversity could be related to this composite metric, rather than to measured $O_{2\left(\mathrm{aq}\right)}$ concentrations, which themselves are also imperfect indicators of redox conditions at depth.

Dissolution rates (*r* in mol m^−2^ s^−1^) were calculated based on the general equation (Lasaga 1995):

(4) $$r=k\times {\left(a_{H^{+}}\right)}^{n_{H^{+}}}\times \exp\left(-\frac{E_{a}}{\mathrm{RT}}\right)\times f\left(\Delta G\right)$$

where *k* is the rate constant (in mol m^−2^ s^−1^), $a_{H^{+}}$ is the proton activity, $n_{H^{+}}$ is the reaction order with respect to $H^{+}$, *E*_*a*_ is the activation energy (in J mol^−1^), and *f*(ΔG) is a function describing the dependence of the dissolution rate on the Gibbs free energy of the reaction. For basaltic glass, *f*(ΔG) is successfully described using a simple transition state theory (TST)‐based function (Daux et al. 1997). For olivine, although no experimental data exist (Daval et al. 2022), TST was assumed to apply for consistency (i.e., $1-\exp\left(\Delta G/\mathrm{RT}\right)$). For olivine, the kinetic parameters were taken from Rimstidt et al. (2012), valid for pH > 5.6 and temperature between 0°C and 150°C (*E*_*a*_ = 66 kJ mol^−1^, $n_{H^{+}}$ = 0.26, $k=10^{4.07}$ mol m^−2^ s^−1^). For basaltic glass, the kinetic parameters were derived from the work of Daux et al. (1997) and Wolff‐Boenisch et al. (2011) (*E*_*a*_ = 41 kJ mol^−1^, $n_{H^{+}}$ = −0.39, *k* = 3.57 × 10^−5^ mol m^−2^ s^−1^).

## 3 Results

### 3.1 Main Physical and Chemical Characteristics of the Sampled Groundwaters

Field measurements and groundwater chemical analyses are summarised in Table 1.

**TABLE 1:** Characteristics of the geothermal groundwaters sampled in March (M22‐i) and October (O22‐i) 2022 in the wells shown in Figure 1.

Sample	*T* (°C)	pH (~20°C)	Cond. (mS cm 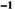 )	$O_{2\left(\mathrm{aq}\right)}$ (ppm)	DOC (mg L 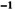 )	TN (mg L 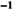 )	$H_{2}S$ (ppm)	$SiO_{2\left(\mathrm{aq}\right)}$ (ppm)	B (ppm)	$Na^{+}$ (ppm)	$K^{+}$ (ppm)	$Ca^{2+}$ (ppm)	$Mg^{2+}$ (ppm)	$Al^{3+}$ (ppm)	Fe (ppm)	$Cl^{-}$ (ppm)	$F^{-}$ (ppm)	${\mathrm{CO}}_{2}$ (ppm)	${{\mathrm{SO}}_{4}}^{2-}$ (ppm)
M22‐1^a^	109	8.3	7.15	3.2	0.32	n.d.^c^	0.09	102	0.192	734	21	676	0.776	0.019	0.098	2054	0.67	4.5	252
M22‐2^a^	98	8.4	4.71	2.1	0.68	n.d.	0.18	111	0.180	576	17	405	0.627	0.020	0.079	1491	0.74	5.5	188
M22‐3^b^	93	9.9	0.38	2.7	0.41	n.d.	0.26	108	0.028	45	1.35	2.6	0.001	0.161	0.001	22	0.42	26.4	14
M22‐4	84	9.6	0.23	2.9	0.38	n.d.^c^	0.03	46	0.012	42	0.73	3.5	0.013	0.159	0.002	30	0.16	25.7	12
M22‐5	70	9.7	0.18	3.9	0.29	n.d.	0.14	49	0.018	33	0.49	2.7	0.003	0.099	0.057	15	0.26	21.2	12
M22‐6^b^	71	9.8	0.22	2.9	0.29	n.d.	0.38	59	0.029	41	0.65	3.7	0.015	0.097	0.009	18	0.46	31	17
M22‐7	87	8.8	0.90	2.2	0.16	n.d.	0.57	99	0.175	155	4.76	15	0.016	0.018	0.002	207	1.90	21.6	64
M22‐8	66	9.3	0.42	3.7	0.33	n.d.	1.15	102	0.255	80	2.04	3.9	0.008	0.027	0.006	48	2.94	32.7	51
M22‐9	59	9.8	0.54	4.0	0.28	n.d.	0.29	68	0.156	93	1.58	7.9	0.009	0.044	0.003	95	2.58	10.1	53
M22‐10^a^	110	10.0	0.29	2.2	0.09	n.d.	0.34	142	0.048	61	2.32	2.9	0.007	0.215	0.005	43	0.73	17.7	19
M22‐11^a^	96	9.8	0.45	2.2	0.19	n.d.	0.15	106	0.250	98	1.86	2.8	0.016	0.118	n.d.	49	0.92	20.1	67
O22‐1	71	10.6	0.27	0.5	0.40	< 0.1	0.14	113	0.083	50	0.85	2.3	0.015	0.354	0.012	10	1.04	13.4	73
O22‐2	67	10.6	0.27	0.6	0.13	< 0.1	0.20	120	0.083	52	0.79	2.0	0.017	0.355	0.034	10	0.99	12.7	71
O22‐3	64	11.0	0.26	0.8	0.22	< 0.1	0.10	82	0.083	44	0.56	2.3	0.022	0.549	0.02	5	0.71	11.7	40
O22‐4	40	10.2	0.19	0.6	0.62	< 0.1	0.07	74	0.062	41	0.21	2.4	0.010	0.018	n.d.	n.d.	n.d.	19.1	n.d.
O22‐5	65	10.6	0.27	0.7	0.23	< 0.1	0.14	90	0.285	50	0.59	2.4	0.070	0.371	0.018	18	1.50	20.4	57
O22‐6	64	10.3	0.30	n.d.	n.d.	n.d.	0.14	79	0.206	53	0.90	3.7	0.091	0.298	0.013	18	1.40	12.5	139
O22‐7	58	9.9	0.27	0.6	0.32	< 0.1	0.44	130	0.454	71	2.05	2.4	0.032	0.370	0.022	14	1.04	31.1	160
O22‐8^a^	89	10.4	0.38	0.8	1.47	1.16	0.17	75	0.188	49	0.81	3.5	0.032	0.317	0.012	28	2.20	11.8	121
O22‐9	30	9.1	0.47	0.7	0.29	< 0.1	0.10	46	0.129	81	3.71	6.7	0.312	0.258	0.011	20	0.10	50.9	151
O22‐10	87	9.6	0.36	0.4	0.61	< 0.1	0.22	164	0.252	69	2.37	3.2	0.061	0.347	0.024	18	1.10	45	163
O22‐11	65	7.0	1.52	0.5	0.23	< 0.1	n.d.	192	0.223	n.d.	10	23	0.227	0.027	0.587	n.d.	n.d.	n.d.	n.d.
Mean	75	9.7	0.91	1.8	0.38	—	0.25	98	0.150	120	3.48	53.6	0.110	0.190	0.050	211	1.09	21.2	86
Min	30	7.0	0.18	0.4	0.09	—	0.03	46	0.012	33	0.21	2.0	0.001	0.018	0.001	5	0.10	4.5	12
Max	110	11.0	7.15	4.0	1.47	—	1.15	192	0.454	734	21	676	0.776	0.549	0.587	2054	2.94	50.9	252
Median	71	9.8	0.33	2.1	0.29	—	0.17	101	0.166	53	1.47	3.4	0.017	0.160	0.013	21	0.96	20.1	66
Q1	64	9.4	0.27	0.6	0.23	—	0.14	74	0.067	45	0.75	2.5	0.011	0.031	0.006	17	0.62	12.5	35
Q3	89	10.3	0.47	2.9	0.40	—	0.29	113	0.219	81	2.36	6.0	0.068	0.340	0.027	48	1.43	26.4	142
IQR	24	0.9	0.20	2.3	0.17	—	0.15	38	0.152	36	1.61	3.6	0.057	0.308	0.021	31	0.81	13.9	107

*Note:* Temperature (*T*), pH (measured at ~20°C), conductivity (Cond.), and dissolved O_2_ ($O_{2\left(\mathrm{aq}\right)}$) concentrations were measured in the field at the wellhead at ~20°C. For ${\mathrm{CO}}_{2}$ and H_2_S concentrations as well as dissolved organic carbon (DOC) and total nitrogen (TN), see Section 2. Statistical data are also provided (i.e., mean, minimum (Min), maximum (Max), median, first quartile (Q1), third quartile (Q3) values, as well as interquartile ranges (IQR = Q3−Q1)).

^a^ Samples for which PCR amplification of the 16S rRNA gene with domain‐specific primers failed for bacteria and archaea (see also Table S1).

^b^ Samples for which PCR amplification of the 16S rRNA gene with domain‐specific primers was successful for bacteria but not for archaea (see also Table S1).

^c^ Not determined (measurement failed).

Temperatures at the wellhead ranged from 30°C to 110°C, with half of them comprised between 58°C and 71°C. Groundwater pH values ranged from 7.0 to 11.0. Only well O22‐11 exhibited near‐neutral pH. Three wells (i.e., M22‐1, M22‐2 and M22‐7) showed slightly basic pH with values between 8 and 9. All of the other wells exhibited pH values above 9. These measurements refer to pH measured at ~20°C unless stated otherwise; they do not necessarily reflect in situ (high‐temperature) conditions, which may differ significantly due to thermal and chemical gradients in the subsurface.

Most groundwater samples exhibited consistent conductivity values (0.18–0.90 mS cm^−1^), typical of freshwater. Exceptions included wells M22‐1 (7.15 mS cm^−1^), M22‐2 (4.71 mS cm^−1^), and O22‐11 (1.52 mS cm^−1^), with M22‐1 and M22‐2 also showing markedly elevated concentrations of $Na^{+}$, $Ca^{2+}$, $Cl^{-}$, and, to a lesser extent, $K^{+}$, $Mg^{2+}$, and ${{\mathrm{SO}}_{4}}^{2-}$, consistent with their higher conductivity. These two wells also had the lowest ${\mathrm{CO}}_{2}$ contents (4.5 and 5.5 ppm). ${\mathrm{CO}}_{2}$ concentrations varied widely (4.5–50.9 ppm), as did dissolved $SiO_{2}$ ($SiO_{2\left(\mathrm{aq}\right)}$), which nevertheless showed consistently high values overall (46–192 ppm), reflecting significant weathering of the host rocks. Regarding DOC and TN, well O22‐8 stood out for higher levels (1.47 and 1.16 ppm), while other wells had DOC content between 0.09 and 0.68 ppm and TN < 0.1 ppm. Finally, $O_{2\left(\mathrm{aq}\right)}$ levels (measured at ~20°C) ranged from 0.4 to 4.0 ppm.

**FIGURE 2:**
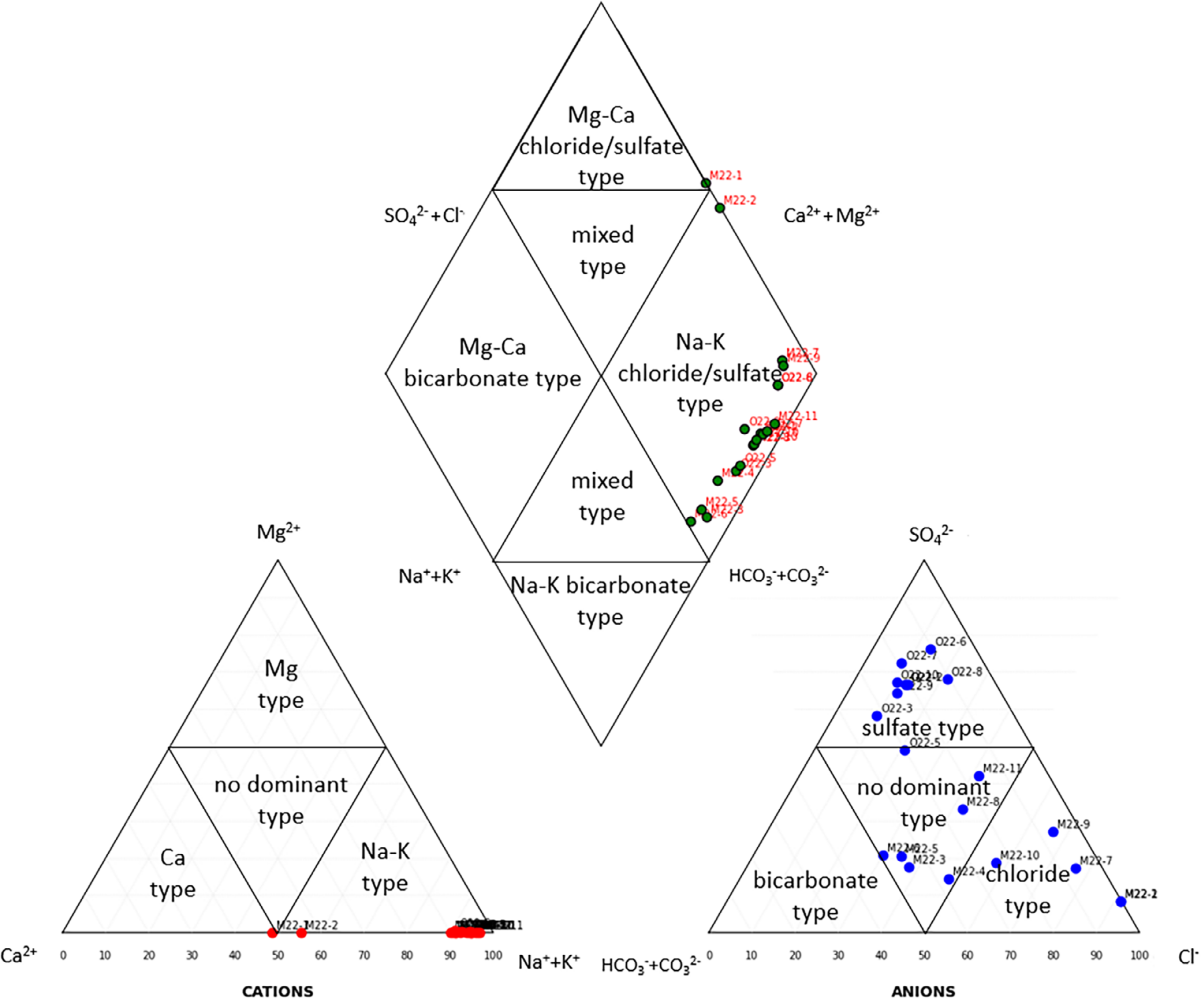
Piper diagram showing the major ion composition of groundwater samples, overall reflecting hydrochemical facies. The cation and anion triangles illustrate relative concentrations of $Na^{+}$, $K^{+}$, $Ca^{2+}$, $Mg^{2+}$, $Cl^{-}$, ${{\mathrm{SO}}_{4}}^{2-}$, and ${{\mathrm{HCO}}_{3}}^{-}$ (Table 1). Samples cluster into distinct groups, suggesting variable water–rock interactions and groundwater sources.

To help understand the water chemistry of the sampled wells, the concentrations of major ions were plotted in a Piper diagram (Figure 2). The integrated analysis of chemical signatures and geographic distribution (Figure 1) led to the identification of three main geochemical groups of geothermal wells, mainly through the analysis of the anion triangle of the Piper diagram. It shows clear spatially coherent patterns that reflect both fluid–rock interaction processes and source characteristics. The first group includes wells such as M22‐1 and M22‐2, which cluster in the $Na^{+}$–$Cl^{-}$ field and exhibit the highest total salinity. These wells, located in the Seltjarnarnes peninsula, have residual marine signatures from seawater ingress in coastal fractured basalts. A second group, represented by wells such as O22‐6, O22‐7, and O22‐8, plots closer to the ${{\mathrm{SO}}_{4}}^{2-}$ corner of the anion triangle of the Piper diagram, with intermediate salinity. These wells are located mainly in northern Iceland and likely reflect mixing between shallow meteoric water and deeper evolved fluids, along with variable degrees of basalt weathering and ${\mathrm{CO}}_{2}$ input from biological or magmatic sources. Notably, despite moderate alkalinity, none of the groundwater samples falls in the ${{\mathrm{HCO}}_{3}}^{-}$‐dominant corner, reflecting limited mixing with shallow waters of meteoric origin, and indicating a common evolution toward ${{\mathrm{SO}}_{4}}^{2-}$ composition. Finally, a third, more dilute group, lies closer to the center of the anion triangle of the Piper diagram. These wells correspond to southern lowland sites and may tap fluids with shorter residence times, and/or low‐temperature alteration pathways. Overall, no samples exhibit the low pH and high sulfate values typical of acid‐sulfate waters. Instead, geochemical trends suggest dominant interaction with basaltic host rocks under reducing to mildly oxidising conditions.

### 3.2 Thermodynamic Status of Groundwaters and Associated Silicate Reactivity

Given that geochemical trends indicate dominant interaction with basaltic host rocks (Figure 2), the thermodynamic favorability of silicate dissolution was assessed by calculating Gibbs free energy for two representative Fe‐bearing silicate phases common in basaltic environments: forsterite olivine and basaltic glass (Table 2). The ΔG values for forsterite ranged from −38.0 (well M22‐7) to −0.9 kJ mol^−1^ (wells O22‐3 and O22‐5), with a mean of −18.8 ± 10.4 kJ mol^−1^, indicating significant variability in reactivity across the sampled sites. In contrast, basaltic glass exhibited a narrower range of ΔG values, with a mean of −13.5 ± 2.3 kJ mol^−1^.

**TABLE 2:** Main thermodynamic parameters estimated for the collected groundwater samples and associated potential reactivity of forsterite olivine (Fo) and basaltic glass (BG),i.e., Gibbs free energy ΔG, dissolution rate *r*, redox potential $E_{H}$ and pH at the temperature of the groundwater measured at the wellhead (pH). When calculations could not be performed due to missing data, dissolution was assumed to occur under far‐from‐equilibrium conditions (i.e., $f\left(\Delta G\right)=1$; see also Section 2).

Well	ΔG(Fo) (kJ mol^−1^)	ΔG(BG) (kJ mol^−1^)	pH 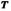	$E_{H}$ (V)	*r*(Fo) (mol m^−21^ s^−1^)	*r*(BG) (mol m^−2^ s^−1^)
M22‐1	−24.8	−15.1	7.3	−0.31	1.26 × 10 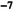	6.22 × 10 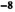
M22‐2	−25.4	−13.6	7.5	−0.32	6.03 × 10 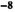	5.08 × 10 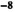
M22‐3	−26.1	−15.0	8.8	−0.45	2.06 × 10 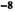	1.36 × 10 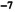
M22‐4	−20.5	−15.6	8.7	−0.42	1.26 × 10 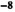	8.85 × 10 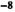
M22‐5	−26.8	−15.0	9.1	−0.44	4.00 × 10 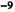	7.21 × 10 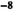
M22‐6	−17.4	−14.8	9.0	−0.43	4.53 × 10 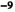	6.88 × 10 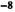
M22‐7	−38.0	−13.6	7.9	−0.35	2.46 × 10 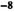	4.84 × 10 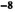
M22‐8	−32.3	−12.1	8.7	−0.40	3.86 × 10 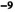	4.25 × 10 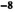
M22‐9	−25.4	−13.4	9.1	−0.42	1.85 × 10 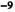	4.48 × 10 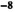
M22‐10	−9.5	−16.0	8.7	−0.46	5.47 × 10 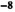	2.26 × 10 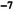
M22‐11	−12.4	−15.7	8.8	−0.44	2.42 × 10 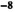	1.52 × 10 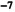
O22‐1	−11.4	−12.7	9.3	−0.45	3.73 × 10 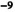	9.00 × 10 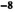
O22‐2	−10.2	−12.4	9.4	−0.45	2.65 × 10 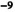	8.32 × 10 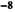
O22‐3	−0.9	−15.9	9.9	−0.49	4.33 × 10 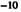	1.15 × 10 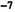
O22‐4	n.d.^a^	n.d.	9.7	n.d.	3.00 × 10 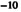	3.12 × 10 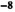
O22‐5	−0.9	−13.6	9.5	−0.46	5.89 × 10 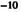	8.35 × 10 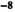
O22‐6	n.d.	n.d.	9.6	−0.46	1.96 × 10 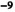	8.75 × 10 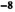
O22‐7	−15.3	−9.9	9.3	−0.43	1.52 × 10 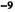	5.13 × 10 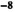
O22‐8	n.d.	n.d.	9.4	−0.48	1.13 × 10 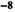	2.01 × 10 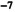
O22‐9	−27.6	−7.2	9.0	−0.35	1.97 × 10 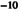	9.88 × 10 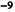
O22‐10	−12.7	−11.2	8.6	−0.41	1.60 × 10 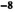	9.08 × 10 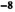
O22‐11	n.d.	n.d.	6.3	n.d.	1.52 × 10 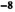	4.72 × 10 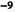

^a^ n.d.: not determined because input data were lacking.

Estimated redox potentials were relatively uniform among the groundwaters sampled, ranging from −0.49 (well O22‐3) to −0.31 V (well M22‐1), with an average value of −0.42 ± 0.05 V (Table 2). These values are indicative of reducing conditions, assuming that the redox potential of the groundwaters was controlled by redox equilibrium between H_2_S and ${{\mathrm{SO}}_{4}}^{2-}$ species.

**FIGURE 3:**
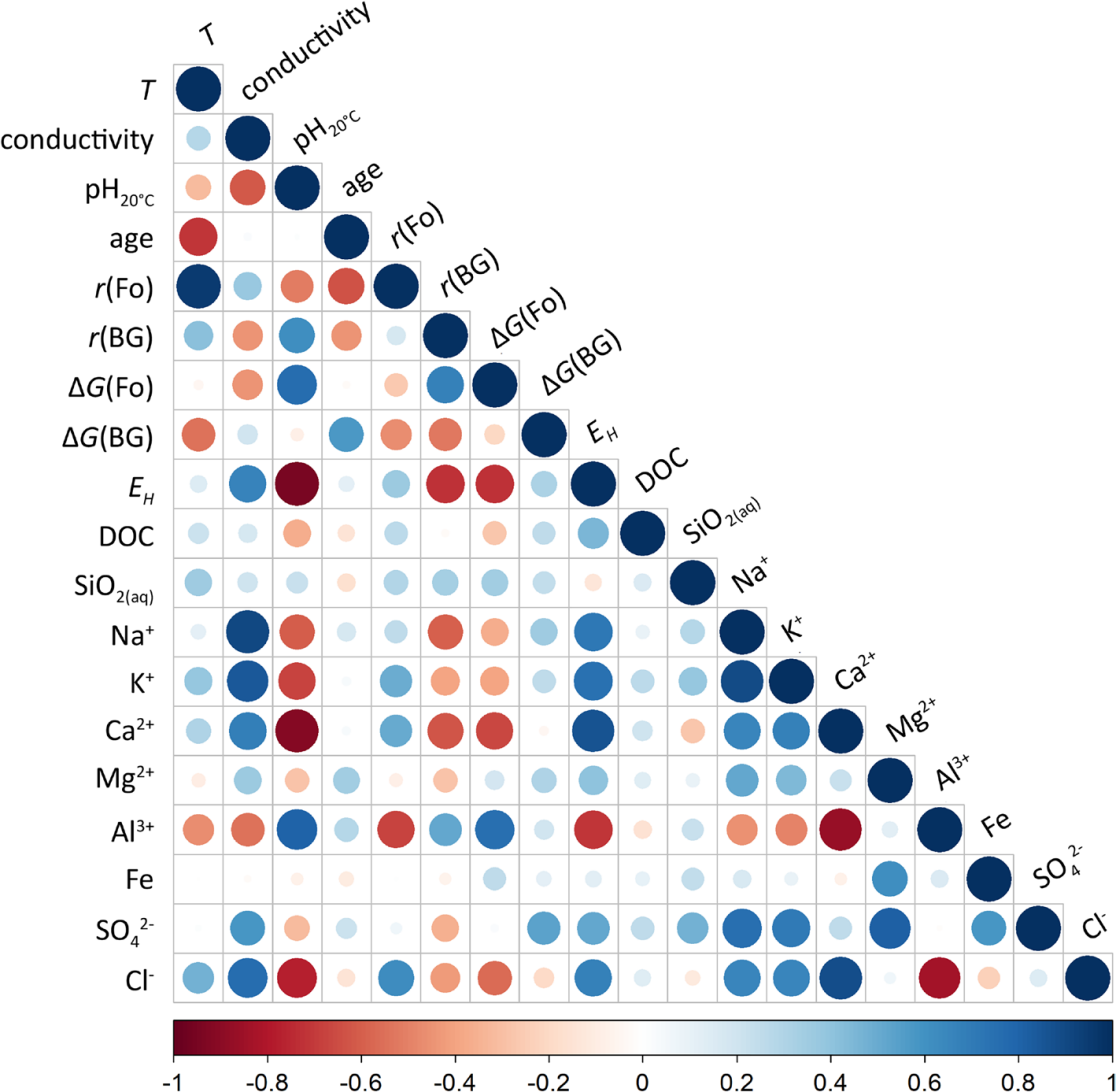
Correlation matrix of groundwater physical and chemical characteristics. Colours represent Spearman correlation coefficients ρ, with positive values shown in blue and negative values in red. *T* (°C) denotes groundwater temperature at the wellhead, pH_20°C_ is the pH measured at ~20°C, age is the mean bedrock age in Myr, *r*(Fo) and *r*(BG) are the dissolution rates of forsterite and basaltic glass, respectively, while ΔG(Fo) and ΔG(BG) are the corresponding Gibbs Free energy of dissolution; $E_{H}$ is the calculated redox potential (Tables 1 and 2; Table S1). Elemental concentrations (Table 1) are included without excluding any wells.

Silicate dissolution being a possible source of nutrients and electron donors—key factors in sustaining subsurface microbial ecosystems (e.g., Henri et al. 2016)—the potential reactivity of rock‐forming phases was assessed by calculating their dissolution rates in each sampled well under in situ groundwater conditions (Table 2). Forsterite dissolution rates ranged from 1.97 × 10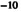 to 1.26 × 10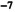 mol m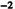 s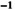, with a mean value of (1.78 ± 2.93) × 10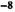 mol m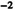 s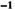. Basaltic glass dissolution rates were generally higher, ranging from 4.72 × 10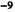 to 2.26 × 10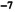 mol m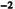 s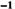, with a mean value of (8.36 ± 5.53) × 10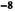 mol m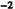 s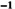 (Table 2). Temperature emerged as the dominant control on forsterite reactivity, exhibiting strong Spearman correlation coefficients (ρ = 0.92, *p* < 0.001; Figure 3). Consistently, the lowest calculated forsterite reactivity occurred in well O22‐9, which also showed the lowest temperature (i.e., 30°C; Table 1). In contrast, basaltic glass reactivity was equally influenced by pH (ρ = 0.54, *p* = 0.010) and temperature (ρ = 0.58, *p* = 0.008) (Figure 3). The minimum reactivity for basaltic glass was observed in well O22‐11, which had the lowest groundwater pH value (pH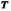 = 6.3). Of note, forsterite and basaltic glass reactivities were calculated to reach maximum values in groundwaters sampled from wells M22‐1 and M22‐10, respectively.

### 3.3 Correlations Between Environmental Parameters

Several significant correlations were detected among the measured environmental parameters and the calculated ones (Figure 3). These interdependencies must be taken into account before evaluating the individual contribution of each parameter to microbial community structure (see Sections 3.4.3 and 4.2). Notably, groundwater temperature as measured at the wellhead was strongly negatively correlated with bedrock age (ρ = −0.68, *p* < 0.001), and strongly positively correlated with forsterite dissolution rate (ρ = 0.92, *p* < 0.001). Temperature also showed a weaker positive correlation with basaltic glass dissolution rates (ρ = 0.55, *p* = 0.008), and a negative correlation with the Gibbs free energy of basaltic glass dissolution (ρ = −0.58, *p* = 0.01). As expected, conductivity was strongly influenced by the concentrations of major ions ($Na^{+}$, $Ca^{2+}$, $Cl^{-}$ and $K^{+}$) (ρ ≥ 0.75, *p* < 0.001). These correlations remain robust even when the outlier wells with extreme $Na^{+}$, $Ca^{2+}$, $Cl^{-}$ and $K^{+}$ concentrations (M22‐1 and M22‐2) were excluded from the dataset. Conductivity values showed weaker correlations with ${{\mathrm{SO}}_{4}}^{2-}$ (ρ = 0.52, *p* = 0.019) and were negatively correlated with pH_20°C_ (ρ = −0.62, *p* = 0.002).

Overall, concentrations of most ions ($Na^{+}$, $K^{+}$, $Ca^{2+}$, $Mg^{2+}$, Fe, ${{\mathrm{SO}}_{4}}^{2-}$ and $Cl^{-}$) were positively correlated with both conductivity and $E_{H}$ values (ρ values ranging from 0.08 to 0.70), but negatively correlated with pH_20°C_ values (ρ values ranging from −0.82 to −0.14), except for $Al^{3+}$, which showed the opposite trend (ρ = −0.68 and *p* = 0.001 for $E_{H}$; ρ = 0.71 and *p* < 0.001 for pH_20°C_). Generally, concentrations were positively correlated with one another, except for $Al^{3+}$, which was inversely correlated with the other elements.

### 3.4 Microbial Diversity and Community Composition Across Sampled Wells

DNA concentrations ranged between ~0.01 (well O22‐3) and ~1.30 ng μL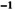 (well O22‐9), and were below the detection limit for six wells (M22‐1, M22‐3, M22‐7, M22‐11, O22‐6 and O22‐8; see details in Table S1). Accordingly, PCR amplification of the 16S rRNA gene (V4‐V5 region) using domain‐specific primers was only partially successful (Table S1). Of the 22 groundwater samples tested, five did not yield bacterial amplicons (wells M22‐1, M22‐2, M22‐10, M22‐11 and O22‐8), all associated with wellhead temperatures exceeding 89°C. Archaeal 16S rRNA gene amplification failed for the same wells, as well as for two additional groundwater samples (wells M22‐3 and M22‐6). With the exception of well M22‐6, all had wellhead temperatures exceeding 90°C. No amplifiable DNA was recovered from control samples. The analyses revealed diverse and distinct bacteria and archaea communities, which are discussed hereafter.

#### 3.4.1 Alpha Diversity

Alpha diversity, measured as the number of observed ASVs, indicated that bacterial diversity remained generally below 200 ASVs per sample with an average of 147, with notable exceptions such as in wells O22‐7, O22‐9 and M22‐6 (Figure S2A). Bacterial richness decreased with increasing groundwater temperature. For archaea, alpha diversity was lower overall, averaging 70 ASVs per sample (Figure S2B). Sample O22‐11, which showed the lowest pH value of 7.0 (Table 1), showed a markedly reduced diversity.

ANOVA analyses showed that temperature had a statistically weak yet significant influence on bacterial alpha diversity (*p* = 0.006), while other physicochemical factors such as conductivity (*p* = 0.165), pH_20°C_ (*p* = 0.906), DOC (*p* = 0.524), $O_{2\left(\mathrm{aq}\right)}$ (*p* = 0.167) did not exhibit significant impacts, although the age of the bedrock (*p* = 0.040), $E_{H}$ (*p* = 0.031) and sulfate concentration (*p* = 0.015) approached relevance. In contrast to bacteria, archaeal alpha diversity did not correlate with temperature (*p* = 0.255). However, sulfate concentration showed a possible effect (*p* = 0.004).

#### 3.4.2 Microbial Community Composition

**FIGURE 4:**
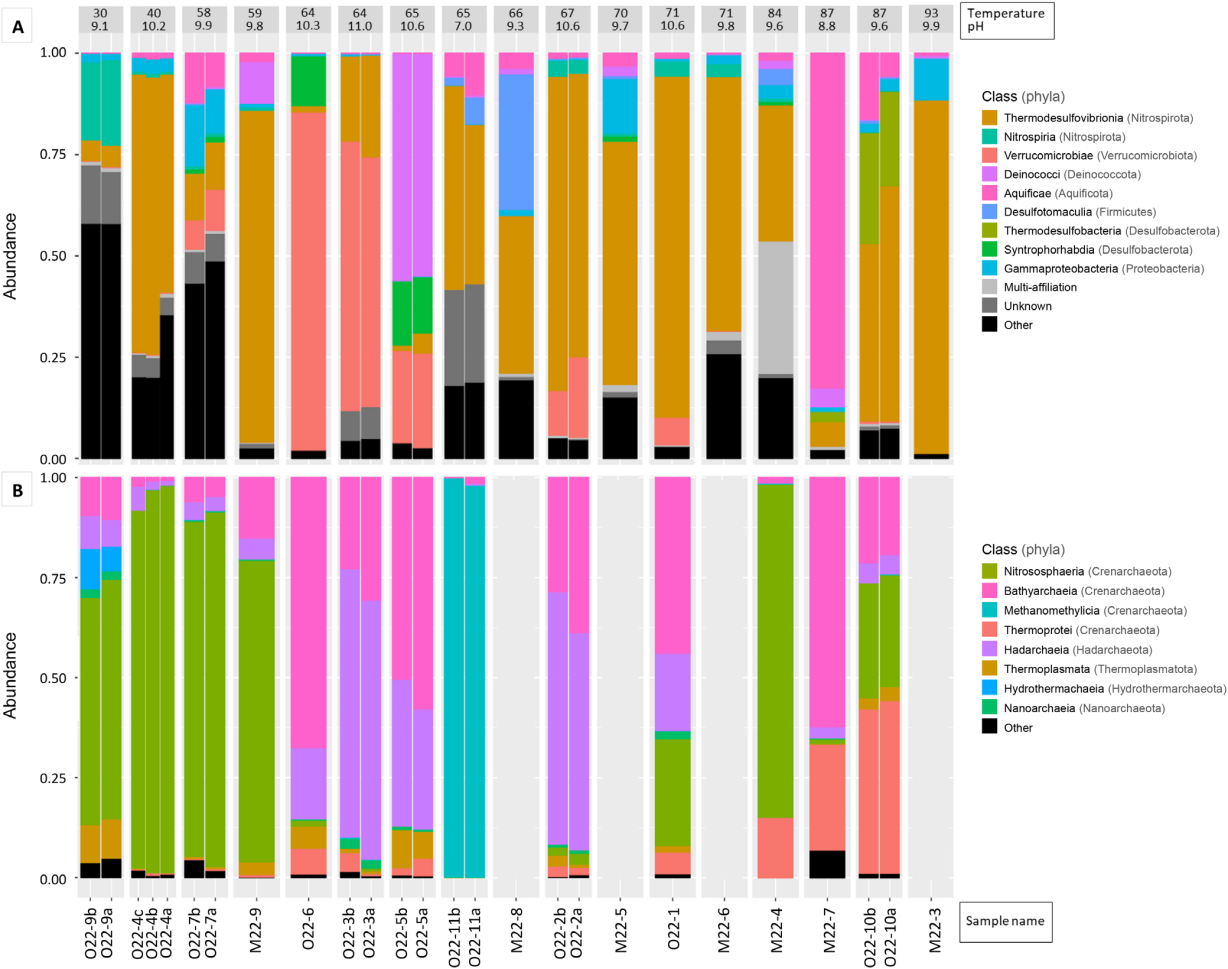
Relative abundance of the most dominant microbial classes retrieved from groundwater samples, shown separately for (A) bacteria (10 most abundant classes) and (B) archaea (8 most abundant classes). Taxonomic assignments were generated using the FROGS pipeline (Escudié et al. 2018) based on the SILVA rRNA gene database (release 138.1; Quast et al. 2012). For each well, the measured wellhead temperature and pH at ~20°C (pH_20°C_) are indicated above the corresponding bar. Samples are ordered by increasing temperature (from 30°C to 93°C for bacteria; and from 30°C to 87°C for archaea). Letters (a, b, c) following sample names denote replicates. See also Figures [Link], [Link].

**FIGURE 5:**
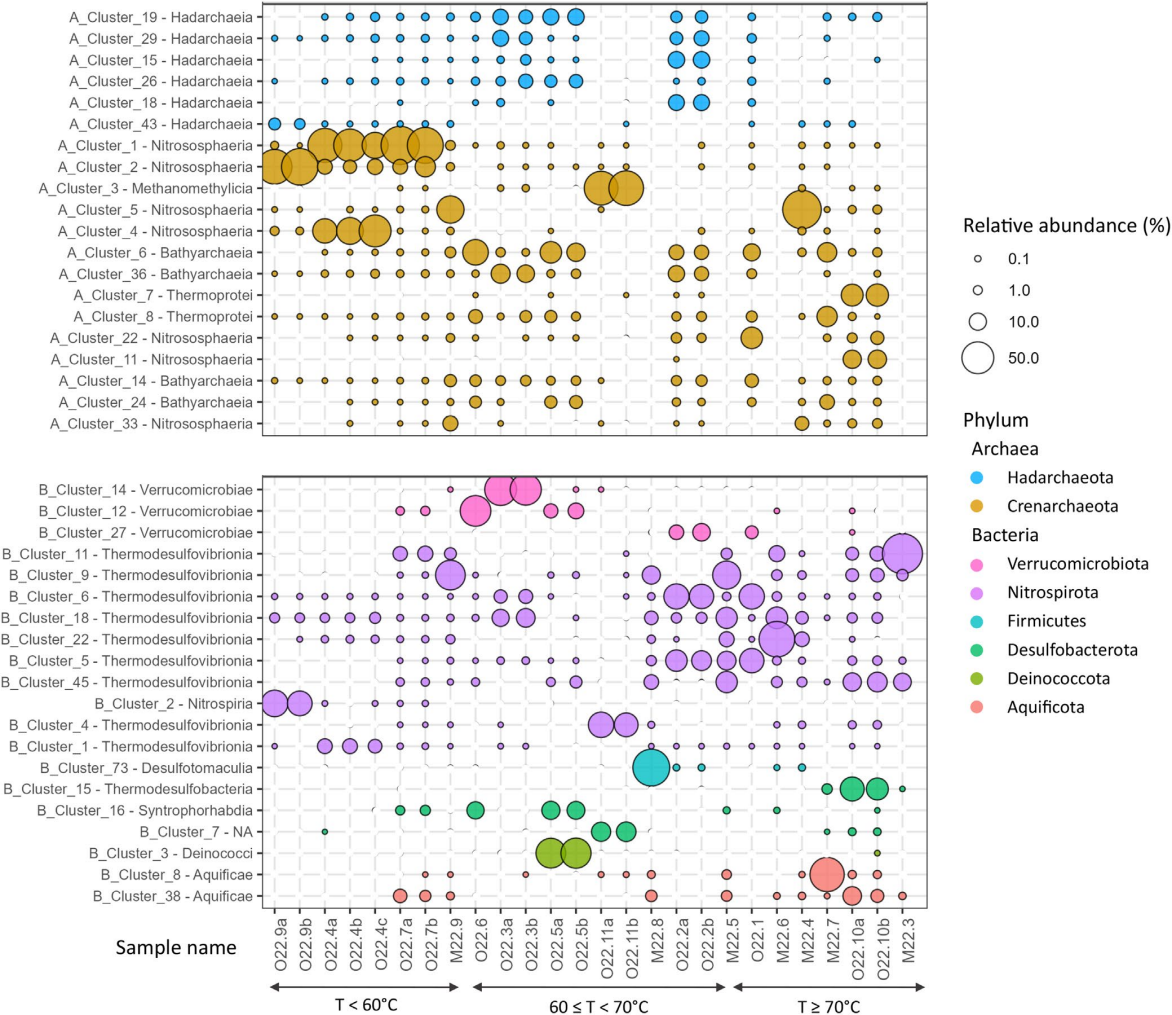
Relative abundance of the 20 most dominant ASVs retrieved from groundwater samples, shown separately for archaea (top; with class name preceded by ‘A’, followed by the corresponding ASV cluster number) and bacteria (bottom; with class name preceded by ‘B’, followed by the corresponding ASV cluster number); NA indicates that no class was identified. Clusters are grouped by phylum which are differentiated by colour. Full taxonomic affiliations of the ASVs are provided in Table S2 for bacteria and Table S3 for archaea. Dot size represents relative abundance (%); white dots are used when the abundance is 0%. Samples are ordered by increasing temperature with temperature ranges indicated below the sample names. Letters (a, b, c) following sample names denote replicates.

Community composition analyses at the phylum (Figure S3), class (Figure 4), genus (Figure S4), and species (Figure S5) levels revealed distinct microbial communities across samples. This was further supported by the distribution of the 20 most abundant ASVs across wells (Figure 5) and associated BLAST analyses (Tables S2 and S3).

For bacteria, Nitrospirota was the most abundant phylum across nearly all samples, representing 40% of all bacterial ASVs. Within this phylum, Thermodesulfovibrionia dominated most wells (1%–87%; Figure 4A), except for O22‐9. Specifically, two ASVs (Cluster_1 and Cluster_6) within Thermodesulfovibrionia were predominant in wells O22‐4, O22‐2 and O22‐1 (40°C–71°C, pH: 10.2–10.6; Figure 5), showing ~99% partial 16S rRNA gene (V4–V5 region) identity to uncultured Thermodesulfovibrionia from an Icelandic basaltic aquifer (Trias et al. 2017) (Table S2). Two other abundant ASVs (Cluster_4 and Cluster_5) were affiliated with the genus *Thermodesulfovibrio*, showing up to 100% identity with sequences from hot springs or mud volcanoes (Hugenholtz et al. 1998; Tobler and Benning 2011). Cluster_4 was found at neutral pH, while Cluster_5 occurred between pH 9.8 and 10.6. Additional dominant Thermodesulfovibrionia ASVs (Cluster_11 and Cluster_9) were detected across wide temperature ranges (58°C–93°C and 59°C–87°C, respectively), but showed only ~95% identity to previously reported environmental sequences. In the cooler well O22‐9 (30°C), an abundant Nitrospirota ASV (Cluster_2) belonged to the class Nitrospiria and was affiliated (100% identity) with *Candidatus* (*Ca*.) *Nitrospira inopinata* (Daims et al. 2015).

Other abundant bacterial phyla included Verrucomicrobiota (13%; particularly dominant in wells O22‐3 and O22‐6 at 58°C–65°C), Deinococcota (4%; notably in well O22‐5 at 65°C), Aquificota (6%; dominating well M22‐7 at 87°C), and Firmicutes (4%; main phylum in wells M22‐8 and M22‐4) (Figure S3A). Within Deinococcota, Cluster_3 showed 100% identity to *Allomeiothermus silvanus* from Icelandic hot springs (Chung et al. 1997). Aquificota was exclusively represented by Aquificae, with dominant ASVs affiliated with *Hydrogenobacter* (Cluster_8 with > 99% identity to *Thermothrix azorensis* ; Odintsova et al. 1996) and *Thermocrinis* (Cluster_38 with 100% identity to *Thermocrinis albus* ; Wirth et al. 2010). Within Firmicutes, Desulfotomaculia was dominant, with Cluster_73 showing 100% identity to *Ca. Desulforudis audaxviator* (Karnachuk et al. 2019). Another abundant ASV (Cluster_15) within Desulfobacterota (class Thermodesulfobacteria) showed 100% identity with *Thermodesulfotobacterium* sp. (Miroshnichenko et al. 2009).

Among archaea, Crenarchaeota was the most abundant phylum, often accounting for up to nearly 100% of archaeal ASVs in several groundwater samples (Figure S3B). Hadarchaeota (0%–67%) and Thermoplasmatota (~3%) were also prevalent.

At the class level (Figure 4B), Crenarchaeota included Nitrososphaeria, Bathyarchaeia, Thermoprotei, and Methanomethylicia. Nitrososphaeria dominated cooler wells (< 60°C). Among Nitrososphaeria, Cluster_2 in well O22‐9 (30°C, pH 9.8) showed 100% identity to *Ca. Nitrosotenuis* (Table S3; Sauder et al. 2018). In wells O22‐4, O22‐7, and M22‐9 (40°C–59°C, pH 9.8–10.2), two Nitrososphaeria ASVs (Cluster_1 and Cluster_5) shared ~99% identity with *Ca. Nitrosocaldus yellowstonensis* (De La Torre et al. 2008). Cluster_5 remained prominent up to 84°C (M22‐4; Figure 5). One Nitrososphaeria ASV (Cluster_4) in O22‐4 was closely related to archaea from a Nevada carbonate spring. Three Nitrososphaeria ASVs (Cluster_22, Cluster_11, Cluster_33), affiliated with *Ca. Caldiarchaeum*, were detected from 59°C to 87°C, with Cluster_11 specific to the hottest sample O22‐11. Thermoprotei increased in abundance in the hottest wells (> 80°C). Among Thermoprotei, Cluster_7 (retrieved in well O22‐10 showing the highest temperature) showed 100% identity to *Saccharolobus solfataricus*/*Sulfolobus islandicus* (Whitaker et al. 2003; Payne et al. 2018; Brock et al. 1972). Bathyarchaeia dominated with Hadarchaeia (Hadarchaeota sole class) in wells with intermediate temperatures (64°C–71°C). The most abundant ASVs in both groups (Cluster_6, Cluster_36, Cluster_14 and Cluster_24 for Bathyarchaeia and Cluster_19, Cluster_29, Cluster_15, Cluster_26, Cluster_18 and Cluster_43 for Hadarchaeia) were related to sequences from deep groundwaters or hot springs (e.g., Spear et al. 2005; Vick et al. 2010). The archaeal community in well O22‐11 was unique, dominated by Methanomethylicia affiliated with *Ca. Methanomethylicus* (with Cluster_3 showing ~98% identity to *Ca. Methanosuratincola petrocarbonis*; Wu et al. 2025), coinciding with its uniquely low pH.

#### 3.4.3 Beta Diversity

**FIGURE 6:**
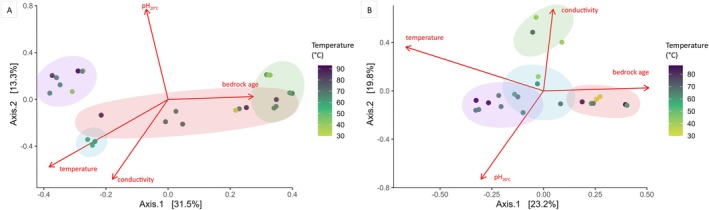
Principal coordinate analysis (PCoA) based on unweighted UniFrac distances for (A) bacterial and (B) archaeal communities in groundwater samples. Coloured ellipses delineate the four phylogenetically distinct groups identified through hierarchical agglomerative clustering (Figure S6). Note that the sampled wells were not clustered identically for bacteria and archaea in the HAC analysis (Figure S6A,B); therefore, the colour codes do not correspond to the same samples in (A) and (B). Point colours represent the temperature at the wellhead (°C).

Beta diversity patterns, assessed through PCoA using unweighted UniFrac distances, revealed well‐separated microbial communities among the sampled wells for both bacteria (Figure 6A) and archaea (Figure 6B), with PCoA groupings supported by clustering analyses (HAC; Figure S6). It is important to note that joint bacterial‐archaeal analyses could not be performed, as amplification of the archaeal 16S rRNA gene was unsuccessful in several wells where bacterial amplification succeeded (i.e., wells M22‐3 and M22‐6) or because the number of archaeal sequences was too low in certain wells for meaningful analysis (i.e., wells M22‐5 and M22‐8) (Table S1).

For bacterial communities, the first axis of the PCoA (accounting for 32% of the variance) was aligned with bedrock age, while the second axis (13%) tracked variation in pH. When PERMANOVA analyses were performed individually for each measured environmental parameter, temperature (14%, *p* = 0.003), DOC (11%, *p* = 0.007), pH (10%, *p* = 0.008) and conductivity (10%, *p* = 0.007) emerged as the most significant drivers of bacterial beta diversity (Table 3). Although the directional vector for bedrock age closely aligned with the first PCoA axis, it accounted for a slightly lower proportion of the variance (9%) and showed weaker statistical support (*p* = 0.022). Two additional variables, namely ${{\mathrm{SO}}_{4}}^{2-}$ (8.5%, *p* = 0.083) and $O_{2\left(\mathrm{aq}\right)}$ (8.2%, *p* = 0.042), accounted for moderate proportions of variance, with only the latter approaching statistical significance. Calculated thermodynamic and kinetic parameters (see Section 3.2) were also tested. Among these, $E_{H}$ (17%, *p* = 0.002), ΔG(BG) (15%, *p* = 0.008), ΔG(Fo) (15%, *p* = 0.003), *r*(BG) (12%, *p* = 0.004), and *r*(Fo) (11%, *p* = 0.004) explained significant fractions of the variance. However, as shown in Figure 3, these composite variables are strongly correlated with measured environmental parameters such as temperature and pH. To avoid collinearity, we chose to retain directly measured environmental parameters rather than derived composite variables in the combined PERMANOVA models. When considering combined drivers, temperature, pH and DOC together accounted for 14% (*p* = 0.001), 10% (*p* = 0.001) and 10% (*p* = 0.005) of the variance in bacterial beta diversity, respectively.

**TABLE 3:** Results of the PERMANOVA analyses quantifying the proportion of variance in bacterial and archaeal beta diversity explained by individual environmental parameters. Variables are ordered by decreasing order of *R*.

Bacteria			Archaea		
Variable	*R* 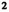	*p*	Variable	*R* 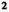	*p*
$E_{H}$	0.17	0.002	ΔG(BG)	0.23	0.001
ΔG(Fo)	0.15	0.003	$E_{H}$	0.20	0.001
ΔG(BG)	0.15	0.008	*T*	0.17	0.001
*T*	0.14	0.003	SO 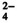	0.17	0.002
r(BG)	0.12	0.004	r(BG)	0.16	0.001
r(Fo)	0.11	0.004	ΔG(Fo)	0.16	0.009
DOC	0.11	0.007	pH_20°C_	0.15	0.002
pH_20°C_	0.10	0.008	r(Fo)	0.13	0.003
Conductivity	0.10	0.007	Bedrock age	0.11	0.015
Bedrock age	0.09	0.022	Conductivity	0.11	0.013
${{\mathrm{SO}}_{4}}^{2-}$	0.085	0.083	$O_{2\left(\mathrm{aq}\right)}$	0.097	0.025
$O_{2\left(\mathrm{aq}\right)}$	0.082	0.042	DOC	0.093	0.034

For archaeal communities, a similar pattern was observed. The first PCoA axis (accounting for 23% of the variance) corresponded roughly to bedrock age and temperature gradients, while the second axis (20%) reflected variation in conductivity and pH. Sample clusters defined by HAC (Figure S6B) did not match those observed for bacteria (Figure S6A), indicating domain‐specific structuring patterns. PERMANOVA analyses conducted on individual measured environmental parameters identified temperature (17%, *p* = 0.001), ${{\mathrm{SO}}_{4}}^{2-}$ (17%, *p* = 0.002), and pH (15%, *p* = 0.002) as the most significant drivers of archaeal beta diversity (Table 3). Additional parameters with lower, though still notable, explanatory power included conductivity (11%, *p* = 0.013), $O_{2\left(\mathrm{aq}\right)}$ (9.7%, *p* = 0.025) and DOC (9.3%, *p* = 0.034). As with bacterial communities, thermodynamic and kinetic variables were also tested and yielded high explanatory power: ΔG(BG) (23%, *p* = 0.001), $E_{H}$ (20%, *p* = 0.001), *r*(BG) (16%, *p* = 0.001), ΔG(Fo) (16%, *p* = 0.009), *r*(Fo) (13%, *p* = 0.003) and bedrock age (11%, *p* = 0.015). However, many of these variables were strongly correlated with primary environmental parameters such as temperature and pH, complicating causal interpretation. To minimise collinearity, composite variables were excluded from models combining multiple drivers. When considering combined effects, temperature, pH and ${{\mathrm{SO}}_{4}}^{2-}$ concentrations together explained 17% (*p* = 0.001), 15% (*p* = 0.002) and 9% (*p* = 0.032) of the variance in archaeal beta diversity.

Taken together, these results suggest that temperature and pH are the most consistent environmental drivers of microbial beta diversity across both bacterial and archaeal domains. While mineral reactivity parameters also explained substantial variance, their strong correlation with temperature and pH complicates their interpretation as independent structuring factors.

## 4 Discussion

### 4.1 Sampling Representativeness in the Context of Basalt‐Hosted Ecosystems

Our comprehensive survey of 22 deep geothermal wells revealed taxonomically rich and ecologically structured bacterial and archaeal communities. Dominant ASVs across both domains exhibited high sequence similarity to strains or environmental sequences previously recovered from geothermal and basaltic environments, notably in Iceland, underscoring their ecological relevance to such systems.

Bacterial communities were frequently dominated by Thermodesulfovibrionia, including ASVs closely matching those from an Icelandic basaltic aquifer (Trias et al. 2017). The consistent presence of *Thermodesulfovibrio* members—i.e., sulfate‐reducing bacteria adapted to high‐temperature and anoxic environments (Umezawa et al. 2021)—suggests an active sulfur cycling in the mesophilic to hyperthermophilic communities (temperatures > 40°C). This is further supported by the detection of other sulfur‐cycling taxa like Thermodesulfobacteria and Desulfotomaculia (e.g., *Ca*. *Desulforudis audaxviator*; Karnachuk et al. 2019). Their prevalence implies the availability of electron donors such as organic compounds or molecular hydrogen ($H_{2}$), consistent with energy sources expected in deep basalt‐hosted systems (e.g., Chapelle et al. 2002; Ménez 2020).

The phylum Aquificota—represented by the genera *Hydrogenobacter* and *Thermocrinis*—was widespread, particularly in high‐temperature wells. These genera are recognised thermophilic chemolithoautotrophs (Murphy et al. 2013; Keller et al. 2025) commonly found in $H_{2}$‐rich and sulfur‐rich hydrothermal environments, including Icelandic geothermal fields (Tobler and Benning 2011). Their co‐occurrence with archaea like *Ca. Nitrosocaldus* and *Ca. Caldiarchaeum* (e.g., wells M22‐9, O22‐7, O22‐10), is consistent with prior studies in Yellowstone hot springs (Murphy et al. 2013; Colman et al. 2024). Their metabolic versatility, including the ability to oxidise $H_{2}$, and reduced sulfur and iron species, positions them as key players in energy acquisition within subsurface basaltic systems influenced by geothermal fluids. The detection of *Nitrospira* in well O22‐9 indicates potential for nitrite oxidation and possibly complete ammonia oxidation under oligotrophic conditions, consistent with observations in deep aquifers and rock‐hosted microbial ecosystems (Daims et al. 2015; Han et al. 2021).

The unidentified ASVs assigned to Verrucomicrobiae may not truly belong to this phylum, but could instead represent novel, unclassified lineages, as commonly observed in extreme environments (Lopez‐Fernandez et al. 2018; Beaver and Neufeld 2024). This highlights the need for deeper metagenomic investigation to achieve more accurate taxonomic resolution.

Archaeal communities were dominated by uncultivated lineages characteristic of deep and basalt‐reCLUSTERlated geothermal environments. Nitrososphaeria were highly abundant, especially in lower temperature wells (*T* ≤ 59°C) and well M22‐4 (84°C). At cooler temperatures (30°C–58°C), ASVs affiliated with *Ca. Nitrosotenuis* were detected, consistent with the optimal growth temperatures of known ammonia‐oxidising archaea (AOA) strains like *Ca. Nitrosotenuis uzonensis* (Lebedeva et al. 2013). At higher temperatures (up to 84°C), AOA lineages affiliated with *Ca. Nitrosocaldus yellowstonii*, a species known to thrive in neutral to alkaline terrestrial geothermal environments (Colman et al. 2024; Keller et al. 2025; De La Torre et al. 2008; Murphy et al. 2013), became increasingly abundant. The prevalence of these AOA lineages suggests, similar to the bacterial genus *Nitrospira*, a significant role for archaea in the nitrogen cycling within these deep geothermal environments. Other abundant Nitrososphaeria ASVs, including those affiliated with *Ca. Caldiarchaeum* (up to 87°C), highlight the metabolic versatility of these lineages, which include chemoorgano‐ and chemolithotrophic capabilities, likely contributing to their ecological competitiveness in extreme conditions (Nunoura et al. 2011; Balbay et al. 2023).

The increased abundance of Thermoprotei, including *Sulfolobus*/*Saccharolobus*, in the hottest wells (> 80°C) was notable. While these taxa are typically associated with high temperature, low pH, and sulfur‐rich environments (Whitaker et al. 2003; Colman et al. 2024), their presence despite the neutral to mildly basic pH suggests potential localized acidic microenvironments or sampling biases (see Section 4.3). Nevertheless, their consistent association with basaltic geochemistry underscores their ecological relevance in such systems. The presence of Bathyarchaeia and Hadarchaeota, consistent with deep crustal and alkaline groundwater environments (Zhou et al. 2020), further reflects the diverse archaeal adaptation to these settings. Finally, the detection of Methanomethylicia in the low‐pH well O22‐11 aligns with findings in other volcanic groundwater systems (Wu et al. 2025), indicating the potential for methanogenesis.

In summary, dominant ASVs—across both domains—show high sequence similarity to strains or environmental sequences previously recovered from geothermal and basaltic environments, especially in Iceland, supporting their ecological relevance to such systems. The taxonomic affiliations observed here are consistent with those found in deep biosphere environments including fractured aquifers and thermally altered volcanic settings. Notably, several abundant ASVs were related to organisms adapted to low‐energy, anaerobic, thermophilic, and chemically challenging environments—hallmarks of the deep subsurface. This includes, for instance, the dominant Firmicutes ASV affiliated with *Ca. Desulforudis audaxviator*, a model deep biosphere taxon. The co‐occurrence of genera such as *Thermodesulfovibrio*, *Hydrogenobacter*, *Thermocrinis*, *Nitrospira Ca. Nitrosotenuis, Ca. Nitrosocaldus*, *Ca. Caldiarchaeum* and *Sulfolobus* highlights a microbial community adapted to geothermal gradients and basaltic geochemistry, capable of sustaining lithotrophic metabolisms under energy‐limited and chemically variable conditions typifying magmatic degassing and hydrothermal alteration. Gaining a deeper understanding of these potentially key subsurface taxa will help clarify their influence on deep biogeochemical cycles, particularly through comprehensive metagenomic characterisation of their metabolic capabilities.

Beyond their relevance to subsurface biogeochemical cycling on Earth, the microbial assemblages identified across Icelandic basalt‐hosted aquifers also provide valuable analogues for understanding microbial roles in silicate weathering and ${\mathrm{CO}}_{2}$ sequestration under natural conditions. The presence of chemolithotrophic and thermophilic taxa capable of exploiting inorganic energy and carbon sources supports the notion that such communities can contribute to mineral alteration and carbon fixation in situ, thereby influencing the long‐term reactivity and carbon storage potential of basaltic formations targeted for CCSU operations (see also Section 4.4). Such influence has already been observed, with evidence linking the development of autotrophic, iron‐oxidising bacteria following ${\mathrm{CO}}_{2}$ injection to shifts in redox conditions within the aquifer, and consequently, to changes in basalt dissolution kinetics (Trias et al. 2017).

Moreover, these ecosystems offer insights into the limits of life in deep, energy‐limited environments, paralleling those invoked for early Earth and for potential subsurface habitats on other planetary bodies such as Mars (e.g., Stevens and McKinley 1995; Nealson et al. 2005; Rucker et al. 2023). Thus, characterizing these microbial communities not only refines our understanding of biogeochemical processes relevant to carbon cycling and mineral transformation, but also broadens the context of deep biosphere research to planetary and astrobiological perspectives.

### 4.2 Linking Microbial Diversity With Environmental Drivers

Although sampling limitations made it difficult to fully characterize microbial communities in relation to geochemical parameters (see Section 4.3), the observed beta diversity still suggests key structuring factors at the well scale, providing valuable guidance for designing microcosms in subsurface mineral dissolution experiments (Section 4.4). These correlations should not be interpreted as direct causal relationships but rather as indicative of possible environmental patterns that may influence microbial community structures and thus warrant targeted experimental investigations.

Consistent with previous studies (e.g., Sharp et al. 2014; Delgado‐Baquerizo et al. 2018), the main environmental drivers of microbial beta diversity included temperature (explaining between 14% and 17% of variance for bacteria and archaea, respectively) and groundwater pH (inversely correlated with conductivity; 10%–15%). Bedrock age may also play a secondary role (9%–11%). Composite parameters—such as the reactivity of basaltic glass (12%–16%) and olivine (11%–13%), as well as the redox potential (17%–20%)—also appeared to significantly contribute to microbial community structure. However, because these variables are inherently derived from—and strongly correlated with—temperature and/or pH (see Section 3.3), their apparent influence should be interpreted with caution. In contrast, concentrations of sulfate and $O_{2\left(\mathrm{aq}\right)}$ had no significant effect on microbial diversity (*p* ≥ 0.02). Below we briefly review each of these parameters.

Temperature was a significant driver of microbial alpha and beta diversity, as previously observed in hot springs of Yellowstone and Iceland (De León et al. 2013; Podar et al. 2020; Bennett et al. 2020). DNA was not recovered above 89°C, and only one sample out of six yielded bacterial DNA at these extreme temperatures. Furthermore, bacterial alpha diversity showed a continuous decline with increasing temperature, in agreement with previous studies (Bregnard et al. 2023; Sharp et al. 2014). The genus *Thermodesulfovibrio* was detected from 59°C to 93°C, with shifts in ASVs composition suggesting thermal niche specialisation. Other temperature‐linked patterns included *Meiothermus* peaking at 65°C, *Thermus* being detected up to 87°C, and Verrucomicrobiota absent above 80°C. Regarding archaea, the temperature gradient was closely related to the relative abundance of genera within the class Nitrososphaeria. The abundance of Thermoprotei increased with temperature, whereas Hydrothermarchaeia and Hadarchaeia were more prevalent in cooler wells.

pH, which here reflects basalt buffering and magmatic inputs, is often suggested as a driver of microbial community composition across ecosystems (e.g., Delgado‐Baquerizo et al. 2018), including in hot springs of Yellowstone (Colman et al. 2024; Inskeep et al. 2013). In well O22‐11, characterised by the lowest pH values (7.0), the bacterial and archaeal communities were clearly distinct. ASVs from the Desulfobacterota phylum were specific to this condition and co‐occurred with Methanomethylicia ASVs, which were only detected at this neutral pH.

The age of the sampled geological units was also considered as a useful proxy in the context of silicate weathering, as it may reflect the extent of mineral surface alteration by secondary phases and the resulting passivation of reactive silicate surfaces during dissolution (Velbel 1993; Nugent et al. 1998)—notably for basaltic glass (Daux et al. 1997) and primary basalt‐forming minerals (Béarat et al. 2006; Daval et al. 2013)—that affect the bioavailability of nutrients and electron donors sustaining chemosynthetic metabolisms. These include reduced species such as ferrous iron Fe(II) or H_2_ derived from the hydration of Fe(II)‐bearing minerals (e.g., Klein et al. 2020; Ménez 2020) for unaltered basalts or alteration products typical of palagonite rims (e.g., Türke et al. 2015) for more evolved ones. Several studies suggested that prolonged water–rock interactions and mineral alteration influence microbial diversity (Santelli et al. 2008, 2009; Lee et al. 2015), shifting dominant metabolisms from, for example, Fe(II) oxidation in younger rocks (with Fe(II) derived from primary basaltic minerals such as olivine) to hydrogenotrophy in older, more altered (e.g., palagonitized) formations (Türke et al. 2015). This shift likely reflects the gradual formation of secondary, non‐redox‐active minerals such as clays, carbonates, and zeolites, that passivate primary mineral surfaces (Santelli et al. 2009), or the depletion of Fe(II)‐bearing phases over time, thereby diminishing iron availability and favouring hydrogenotrophs (Klein et al. 2009).

While bedrock age appeared here to have a limited influence on microbial community structure, thermodynamic and kinetic considerations may provide deeper insight into how the intensity of water–rock interactions shaped microbial assemblages. Thermodynamics can identify energetically favourable metabolic pathways (e.g., Meyer‐Dombard et al. 2005), but kinetic constraints, such as mineral dissolution rates, provide a more realistic proxy for estimating energy availability in situ (Bach 2016; Cockell 2011). Accordingly, we estimated dissolution rates of olivine and basaltic glass—two potential sources for $H_{2}$—and found that both accounted for a significant portion of the observed variance in microbial beta diversity (Table 3).

Concerning bacteria, the five wells with the highest estimated rates for olivine dissolution (i.e., wells M22‐7, M22‐3, O22‐10, O22‐11 and M22‐4) all hosted *Thermodesulfovibrio* species, whereas these taxa were absent from the five wells with the lowest olivine dissolution rates. However, we recall that olivine reactivity was strongly correlated with *T* (ρ = 0.92; *p* = 0.001; Figure 3), complicating causal interpretations: observed microbial patterns may reflect the influence of olivine reactivity, temperature or both.

Assessing the impact of mineral reactivity on archaeal diversity proved more difficult, given the low alpha diversity observed, which suggests incomplete community recovery. Nonetheless, well O22‐10—drilled into rocks older than 2 Myr—hosted archaeal taxa potentially capable of using Fe(III) minerals as electron acceptors. Several species within the genus *Sulfolobus* including *Sulfolobus solfataricus* P1 (which shared 100% 16S rRNA gene identity with ASVs detected in well O22‐10), have been shown to reduce Fe(III) under anaerobic conditions. *Sulfolobus solfataricus* was later reclassified as *Saccharolobus solfataricus*, following the establishment of a new genus encompassing several sugar‐metabolising archaea, some of which also exhibit iron‐reducing capabilities. This reclassification highlights their metabolic versatility and adaptation to extreme environments (Masaki et al. 2018; Sakai and Kurosawa 2018). Notably, this well also exhibited high dissolution rates for both olivine and basaltic glass, consistent with extensive weathering and the likely formation of Fe(III)‐bearing secondary minerals at depth.

In summary, although sampling limitations (see Section 4.3) and intercorrelations among environmental variables complicate the interpretation of microbial community patterns, our results suggest that, beyond the well‐known influences of pH and temperature, the reactivity of the host rock may also shape subsurface microbial assemblages, establishing a potential coupling between microbial diversity and mineral reactivity.

### 4.3 Sampling Limitations

In an attempt to assess the role of deep ecosystems in silicate weathering experiments related to basaltic subsurface environments, it is crucial to design experiments with relevant representatives of these systems. While our study revealed ecologically structured bacterial and archaeal communities in the sampled geothermal wells, it also highlighted limitations in ecological representativeness due to methodological constraints and the extreme nature of these environments.

First, the inability to obtain bacterial and archaeal amplicons of the 16S rRNA gene from up to seven of the 22 groundwater samples, all associated with wellhead temperatures exceeding 89°C, highlights the challenges of characterising microbiota in very high‐temperature subsurface environments. Even in samples where archaeal 16S rRNA gene amplification succeeded, it sometimes yielded sequences in quantities too low for meaningful analysis (as in wells M22‐5 and M22‐8). These gaps restricted the scope for comprehensive domain‐wide community comparisons and reduced the ecological resolution for high‐temperature or low‐biomass habitats. Such biases in molecular detection may lead to an underestimation of archaeal diversity and compositional shifts, as also supported by the low alpha diversity obtained for archaea (Figure S2B). The dominance of certain bacterial lineages—such as Thermodesulfovibrionia, *Hydrogenobacter*, *Thermocrinis*, *Meiothermus* and *Thermus*—and archaeal lineages—such as *Saccharolobus*, *Ca. Caldiarchaeum*, *Ca. Nitrosotenuis* and *Ca. Nitrosocaldus*—(Figures 4, 5, S4, and S5 and Tables S2 and S3) could reflect both true ecological prominence and selective biases introduced by primer specificity or DNA stability under extreme conditions.

Second, while hierarchical clustering and ordination analyses revealed some patterns in microbial assemblage differentiation (Figures 6 and S6), the ecological interpretation of these clusters should be approached with caution. In addition to the biases mentioned just above, sampling was conducted from wells that vary considerably in terms of infrastructure, from cased and instrumented wells operated by geothermal energy companies to basic farm wells on private land. In particular, sampled wells were neither systematically cased nor equipped with, for example, packers, overall preventing the isolation of discrete depth intervals during sampling. As a result, most collected groundwaters represented a mixture of fluids originating from different depths within the borehole (typically, with one productive fracture responsible for more than half of the water collected at the wellhead). This may reduce the spatial resolution of geochemical and microbial analyses, potentially obscuring depth‐specific signatures and introducing uncertainty in attributing observed patterns to specific in situ conditions. This may first explain the lack of influence found for $O_{2\left(\mathrm{aq}\right)}$ concentrations—all wells were dysoxic (0.4–4.0 mg L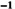), yet aerobes, microaerophiles, and anaerobes co‐occurred in some groundwater samples—although transient recharge events (Bochet et al. 2020), or in situ “dark oxygen” production (e.g., Ruff et al. 2023; Sweetman et al. 2024) cannot be completely ruled out. Similarly, ${{\mathrm{SO}}_{4}}^{2-}$ concentrations did not significantly influence bacterial diversity, despite the presence of sulfate‐reducing *Thermodesulfovibrio* (Sekiguchi et al. 2008). This may also explain the relatively small proportion of variance explained by pH and temperature (on the order of 10% each), while these parameters are well‐identified structuring parameters of microbial diversity (Sharp et al. 2014; Delgado‐Baquerizo et al. 2018; De León et al. 2013; Podar et al. 2020; Bennett et al. 2020; Inskeep et al. 2013; Colman et al. 2024). Another possible explanation, beyond non‐specific sampling, is that the collected samples may have included not only planktonic cells but also biofilm‐associated communities, which are often highly diverse and capable of locally shaping microenvironments. In the subsurface, biofilms are considered the predominant microbial lifestyle, enabling microorganisms to persist in microniches where physicochemical conditions may differ markedly from those measured in bulk groundwater (Flemming and Wuertz 2019). Biofilms also commonly form on pipe walls and well infrastructure. Although flushing procedures were employed to minimise contamination from stagnant water or legacy biofilm, some influence from these sources cannot be entirely ruled out, despite the purge exceeding several volumes of pipe derivations. For example, the abundance of ASVs related to *Ca. Nitrospira inopinata* or *Ca. Nitrosocaldus yellowstonensis* or *islandicus* may be questionable, as *Ca. Nitrospira inopinata* was originally isolated from biofilm growing in hot‐water pipe (Daims et al. 2015; Daebeler et al. 2018), while *Nitrosocaldus* species were enriched from surface biofilms influenced by hot springs (De La Torre et al. 2008). These limitations are not specific to this study, as subsurface research inherently requires adaptation to the constraints imposed by limited accessibility and sampling opportunities, while aseptic sampling remains the exception (Basso et al. 2005; Westmeijer et al. 2024). Moreover, in fractured crustal rocks, fluid flow paths can create localised “hotspots” and transient “hot moments” of microbial activity deep within the subsurface through the mixing of chemically contrasted groundwater (Bochet et al. 2020; Trias et al. 2017), making the temporal dimension and thus the timing of sampling, a critical factor in understanding subsurface microbial dynamics.

### 4.4 Implications for Microcosm Design in Subsurface Mineral Dissolution Experiments

The data presented here demonstrate that microbial communities in basaltic deep aquifers are not only phylogenetically diverse but also highly variable depending on temperature, groundwater chemistry (in particular, pH), and possibly, the host rock reactivity. This heterogeneity poses a challenge for designing broadly representative microcosms, especially when experiments involve varying temperature regimes and distinct pH‐buffering mineral phases. Furthermore, as is common in subsurface environments, a significant proportion of ASVs remains unaffiliated (Figures 4 and [Link], [Link]) or is assigned only to high‐level taxonomic rank, highlighting the considerable unexplored microbial diversity that challenges the design of representative microcosms for experimental studies. It is noteworthy that the coldest groundwaters exhibit the highest alpha diversity but also the greatest proportion of unknown taxa, especially in wells O22‐9 (30°C) and O22‐7 (58°C) (Figures 4 and [Link], [Link]). However, some dominant microbial taxa consistently found across several wells—such as those belonging to the bacterial classes Thermodesulfovibrionia and Aquificae, and the archaeal class Nitrososphaeria—offer promising targets for inclusion in microcosm models due to their apparent ecological relevance and metabolic versatility, provided that they are cultivable and that the experimental conditions used match their niche characteristics (Figure 5). Given the limited number of strains isolated from these environments—for example, Shirokova et al. (2012), Pouder et al. (2025), Chung et al. (1997, 2000) and Kristjánsson et al. (1994), many of which do not represent the dominant lineages highlighted in this study—it can be challenging to artificially reconstruct accurate microcosms. An alternative approach is to use fluids directly sampled from geothermal wells, selecting those that best represent the specific physical and chemical conditions relevant to the experiments or ideally to deploy in situ experimental observatory systems (Orcutt et al. 2011).

## 5 Conclusions

This study provides new insights into the environmental factors shaping bacterial and archaeal diversity in deep basaltic aquifers. Using high‐throughput sequencing and a suite of geochemical measurements and thermodynamic calculations, we showed that microbial beta diversity is primarily structured by temperature and pH, with secondary contributions from bedrock age and, in the case of archaea, from sulfate concentrations. Thermodynamic and kinetic parameters such as fluid saturation and silicate dissolution rates also explained a substantial fraction of the observed variance, but their individual contributions remain difficult to disentangle due to their strong covariation with temperature and pH. Bacterial and archaeal communities exhibited distinct patterns of organisation, with archaea displaying lower diversity and weaker clustering, possibly reflecting incomplete recovery or niche specialisation. The presence of lineages affiliated with hydrogenotrophic and sulfate‐reducing taxa suggests that energy availability from water–rock interactions plays a role in shaping community composition. Taken together, our results highlight the importance of integrating geochemical and microbiological data when investigating subsurface ecosystems. In particular, they support the idea that host rock reactivity, while often overlooked, may play a role in shaping microbial diversity in basaltic systems. This has implications for the design of future experiments seeking to elucidate the relationships between microbial activity, mineral weathering, and biogeochemical cycling in the deep biosphere.

## Author Contributions

**Juliette Bas‐Lorillot:** investigation, writing – original draft, data curation, formal analysis, visualization. **Bénédicte Ménez:** conceptualization, methodology, validation, investigation, writing – review and editing, supervision. **Bastien Wild:** methodology, validation, formal analysis, investigation, writing – review and editing. **Guillaume Borrel:** formal analysis, writing – review and editing. **Manon Le Bihan:** formal analysis, data curation, writing – review and editing. **Andri Stefánsson:** methodology, investigation, resources, writing – review and editing, supervision, funding acquisition. **Jóhann Gunnarsson‐Robin:** investigation, writing – review and editing. **Anna Bríet Bjarkadóttir:** investigation, writing – review and editing. **Sigríður María Aðalsteinsdóttir:** investigation, writing – review and editing. **Delphine Tisserand:** investigation, writing – review and editing. **Damien Daval:** conceptualization, methodology, validation, formal analysis, investigation, writing – original draft, visualization, supervision, project administration, funding acquisition. **Emmanuelle Gérard:** methodology, validation, formal analysis, investigation, data curation, writing – review and editing, supervision.

## Funding

This work was supported by European Research Council, ERC‐2020‐COG Mobidic 101001275.

## Conflicts of Interest

The authors declare no conflicts of interest.

## Supporting information


**FIGURE S1:**, Rarefaction curves for (A) bacterial and (B) archaeal communities across all groundwater samples. The dashed green and blue lines indicate richness values at 18,038 and 6,286 sequences, the thresholds used to rarefy the bacterial and archaeal ASV tables, respectively (see Methods). For clarity, the *x*‐axis (i.e., sample size) was truncated at 50,000 sequences for bacteria and 20,000 sequences for archaea.

**FIGURE S2:** Observed number of ASVs as a function of wellhead temperature (°C) for each groundwater sample in which DNA extraction and PCR amplification of the 16S rRNA gene were successful. Results are shown separately for (A) bacterial and (B) archaeal communities.

**FIGURE S3:** Relative abundance of the most dominant microbial phyla retrieved from groundwater samples, shown separately for (A) bacteria (10 most abundant phyla) and (B) archaea (six most abundant phyla). Taxonomic assignments were generated using the FROGS pipeline (Escudié et al. 2018) based on the SILVA rRNA gene database (release 138.1; Quast et al. 2012). For each well, the measured wellhead temperature and pH at ~20°C (pH_20°C_) are indicated above the corresponding bar. Samples are ordered by increasing temperature (from 30°C to 93°C for bacteria and from 30°C to 87°C for archaea). Letters (a, b, c) following sample names denote replicates.

**FIGURE S4:** Relative abundance of the most dominant microbial genera retrieved from groundwater samples, shown separately for (A) bacteria (30 most abundant genera) and (B) archaea (20 most abundant genera). Taxonomic assignments were generated using the FROGS pipeline (Escudié et al. 2018) based on the SILVA rRNA gene database (release 138.1; Quast et al. 2012). For each well, the measured wellhead temperature and pH at ~20°C (pH_20°C_) are indicated above the corresponding bar. Samples are ordered by increasing temperature (from 30°C to 93°C for bacteria and from 30°C to 87°C for archaea). Letters (a, b, c) following sample names denote replicates. See also Tables S2 and S3 for sequence similarity analyses using the BLAST+ tool (Camacho et al. 2009).

**FIGURE S5:** Relative abundance of the most dominant microbial species retrieved from groundwater samples, shown separately for (A) bacteria (30 most abundant species) and (B) archaea (20 most abundant species. These 20 species represent the only confidently identified taxa across the dataset). Taxonomic assignments were generated using the FROGS pipeline (Escudié et al. 2018) based on the SILVA rRNA gene database (release 138.1; Quast et al. 2012). For each well, the measured wellhead temperature and pH at ~20°C (pH_20°C_) are indicated above the corresponding bar. Samples are ordered by increasing temperature (from 30°C to 93°C for bacteria and from 30°C to 87°C for archaea). Letters (a, b, c) following sample names denote replicates. See also Tables S2 and S3 for sequence similarity analyses using the BLAST+ tool (Camacho et al. 2009).

**FIGURE S6:** Hierarchical agglomerative clustering based on UniFrac distance matrices and the Ward method for (A) bacterial and (B) archaeal community composition. Letters a, b, c following sample names denote replicates. Coloured clusters highlight four phylogenetically distinct groups that have been reported on the PCoA results (Figure 6).

**TABLE S1:** Characteristics and coordinates of the wells sampled (see Figure 1 for locations). Information on wells were retrieved from well‐specific reports deposited in the database operated by the Icelandic National Energy Authority (Orkustofnun; https://www.map.is/os/) and Jóhannesson (2014). Each DNA concentration value corresponds to the mean of three measurements. Where applicable, ranges are shown for wells with replicates. The ‘PCR amplification’ column indicates the outcome of the 16S rRNA gene amplification using domain‐specific primers: ‘+’ denotes successful amplification for both bacteria and archaea; ‘(−)’ indicates failure for archaea only; and ‘–’ means amplification failed for both domains.
**TABLE S2:** Taxonomic classification and closest cultivated (‘Accession (cult.)’) and environmental (‘Accession (env.)’) relatives—along with the environments from which they were retrieved—of the 20 most abundant bacterial ASVs (ranked similarly to Figure 5) identified in groundwater samples and considered for community analysis using the FROGS pipeline (Escudié et al. 2018) (based on sequence similarity analyses using BLAST searches (Camacho et al. 2009) against the NCBI non‐redundant nucleotide database). *Ca*., *Candidatus*.
**TABLE S3:** Taxonomic classification and closest cultivated (‘Accession (cult.)’) and environmental (‘Accession (env.)’) relatives—along with the environments from which they were retrieved—of the 20 most abundant archaeal ASVs (ranked similarly to Figure 5) identified in groundwater samples and considered for community analysis using the FROGS pipeline (Escudié et al. 2018) based on sequence similarity analyses using BLAST searches (Camacho et al. 2009) against the NCBI non‐redundant nucleotide database. *Ca*., *Candidatus*.

## Data Availability

The data that supports the findings of this study are available in the Supporting Information material. All raw reads were submitted to the NCBI Sequence Read Archive under Bioproject ID PRJNA1347816.
