# Supplementary figures and images for: Groundwater Microbial Diversity Associated With Icelandic Basaltic Subsurface Environments
Source: Environ Microbiol Rep. 2025 Nov 30;17(6):e70238. doi: 10.1111/1758-2229.70238 (PMC12665481; doi:10.1111/1758-2229.70238)

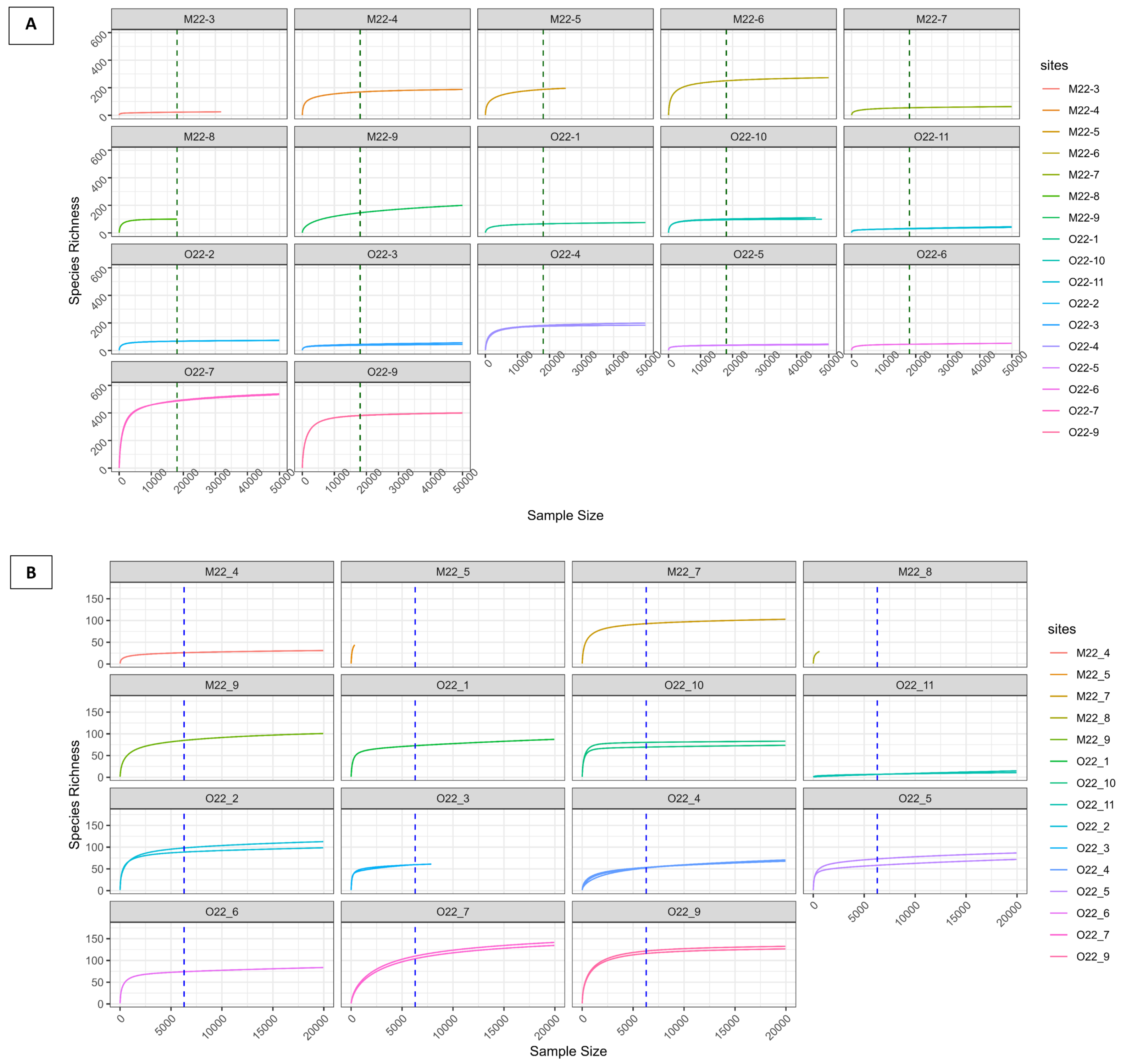

Supplement: Supplementary file 1 — FIGURE S1:, Rarefaction curves for (A) bacterial and (B) archaeal communities across all groundwater samples. The dashed green and blue lines indicate richness values at 18,038 and 6,286 sequences, the thresholds used to rarefy the bacterial and archaeal ASV tables, respectively (see Methods). For clarity, the x‐axis (i.e., sample size) was truncated at 50,000 sequences for bacteria and 20,000 sequences for archaea. [file EMI4-17-e70238-s006.png]

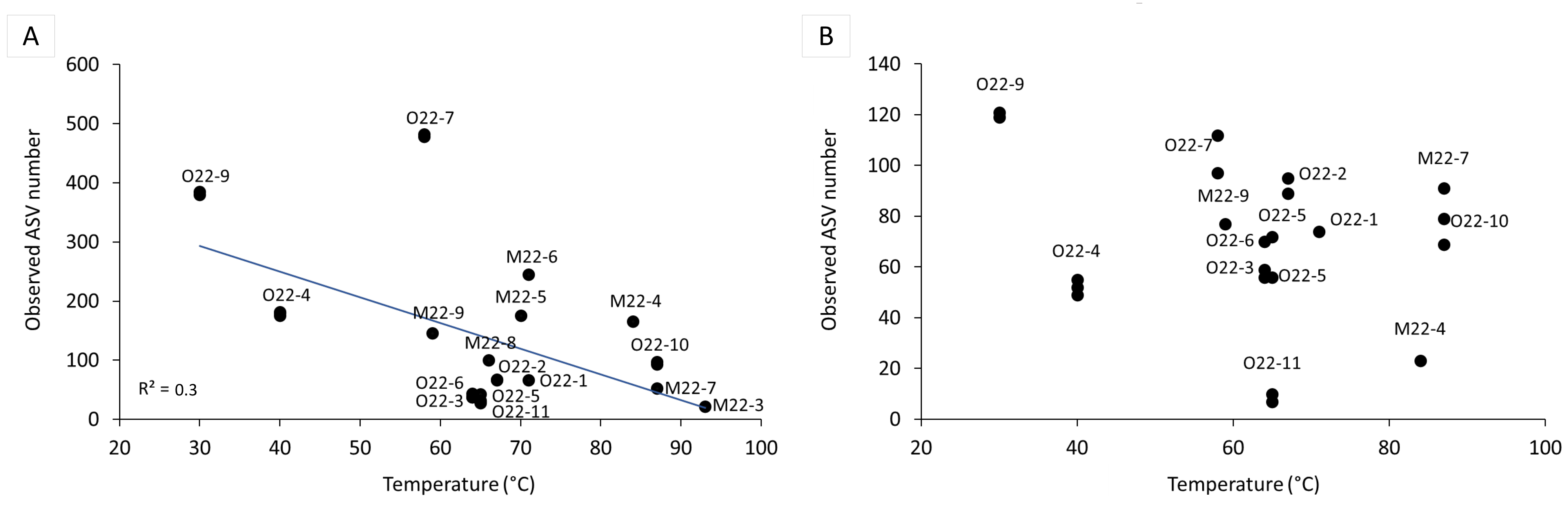

Supplement: Supplementary file 2 — FIGURE S2: Observed number of ASVs as a function of wellhead temperature (°C) for each groundwater sample in which DNA extraction and PCR amplification of the 16S rRNA gene were successful. Results are shown separately for (A) bacterial and (B) archaeal communities. [file EMI4-17-e70238-s005.png]

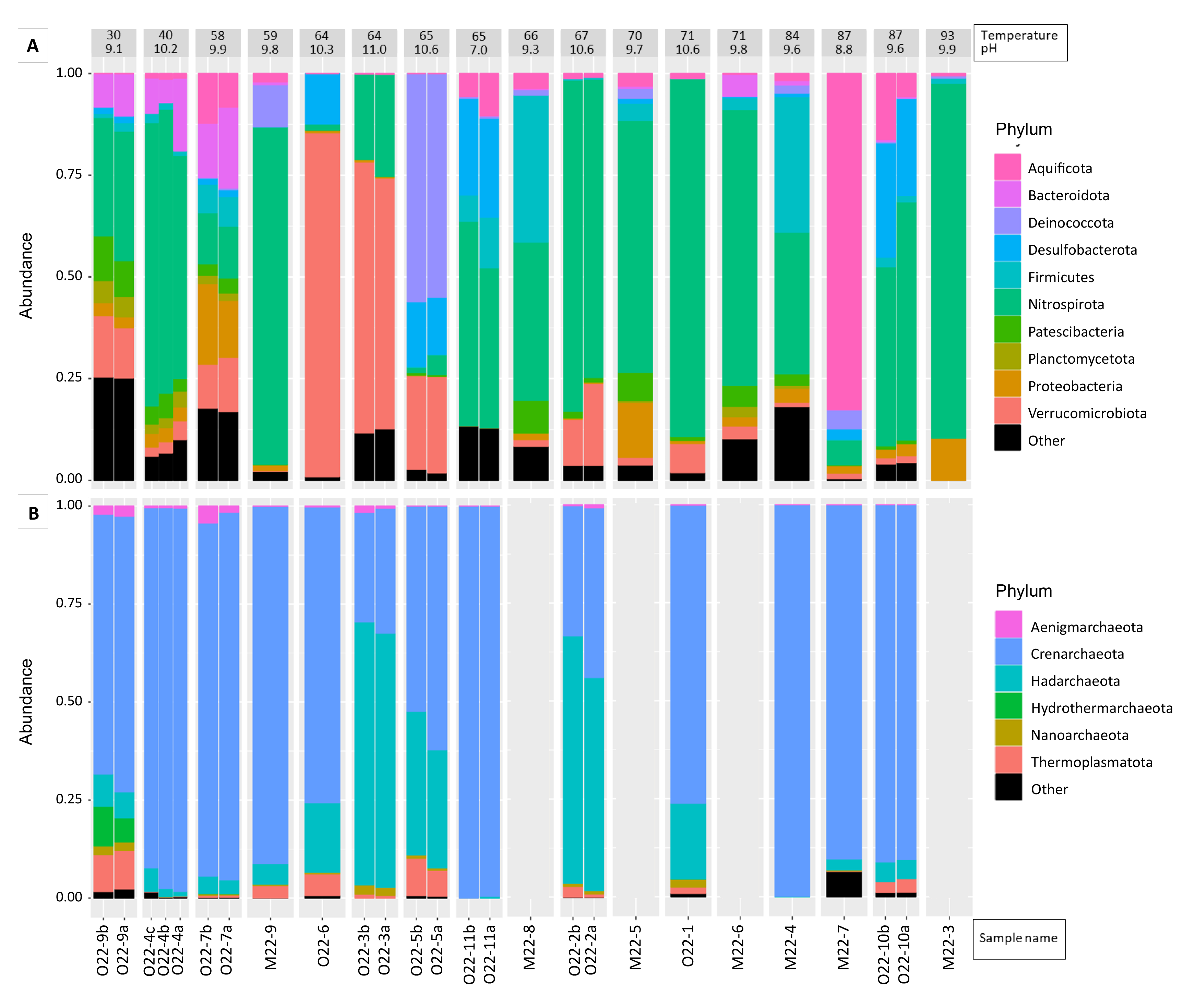

Supplement: Supplementary file 3 — FIGURE S3: Relative abundance of the most dominant microbial phyla retrieved from groundwater samples, shown separately for (A) bacteria (10 most abundant phyla) and (B) archaea (six most abundant phyla). Taxonomic assignments were generated using the FROGS pipeline (Escudié et al. 2018) based on the SILVA rRNA gene database (release 138.1; Quast et al. 2012). For each well, the measured wellhead temperature and pH at ~20°C (pH20°C) are indicated above the corresponding bar. Samples are ordered by increasing temperature (from 30°C to 93°C for bacteria and from 30°C to 87°C for archaea). Letters (a, b, c) following sample names denote replicates. [file EMI4-17-e70238-s003.png]

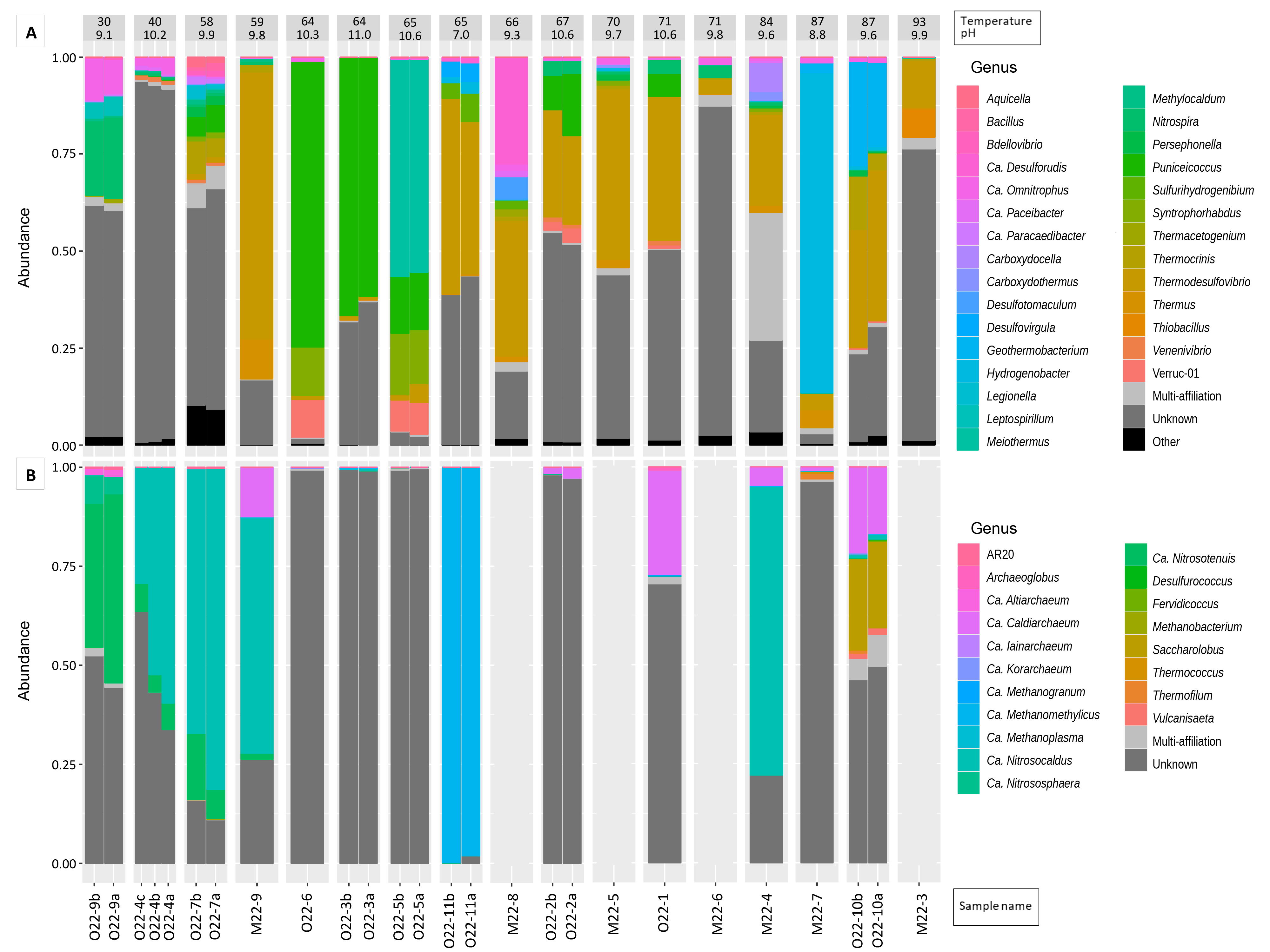

Supplement: Supplementary file 4 — FIGURE S4: Relative abundance of the most dominant microbial genera retrieved from groundwater samples, shown separately for (A) bacteria (30 most abundant genera) and (B) archaea (20 most abundant genera). Taxonomic assignments were generated using the FROGS pipeline (Escudié et al. 2018) based on the SILVA rRNA gene database (release 138.1; Quast et al. 2012). For each well, the measured wellhead temperature and pH at ~20°C (pH20°C) are indicated above the corresponding bar. Samples are ordered by increasing temperature (from 30°C to 93°C for bacteria and from 30°C to 87°C for archaea). Letters (a, b, c) following sample names denote replicates. See also Tables S2 and S3 for sequence similarity analyses using the BLAST+ tool (Camacho et al. 2009). [file EMI4-17-e70238-s001.png]

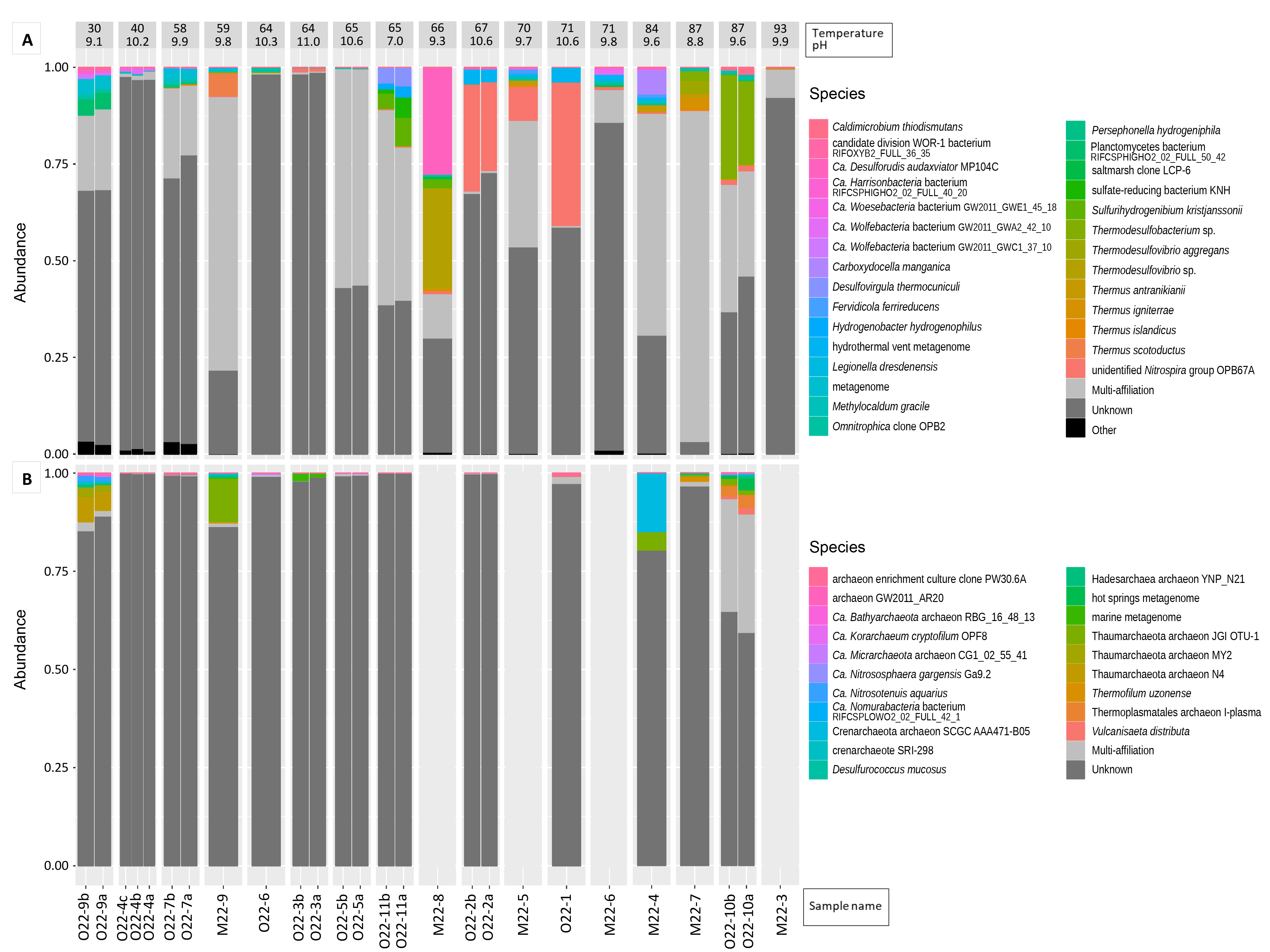

Supplement: Supplementary file 5 — FIGURE S5: Relative abundance of the most dominant microbial species retrieved from groundwater samples, shown separately for (A) bacteria (30 most abundant species) and (B) archaea (20 most abundant species. These 20 species represent the only confidently identified taxa across the dataset). Taxonomic assignments were generated using the FROGS pipeline (Escudié et al. 2018) based on the SILVA rRNA gene database (release 138.1; Quast et al. 2012). For each well, the measured wellhead temperature and pH at ~20°C (pH20°C) are indicated above the corresponding bar. Samples are ordered by increasing temperature (from 30°C to 93°C for bacteria and from 30°C to 87°C for archaea). Letters (a, b, c) following sample names denote replicates. See also Tables S2 and S3 for sequence similarity analyses using the BLAST+ tool (Camacho et al. 2009). [file EMI4-17-e70238-s004.png]

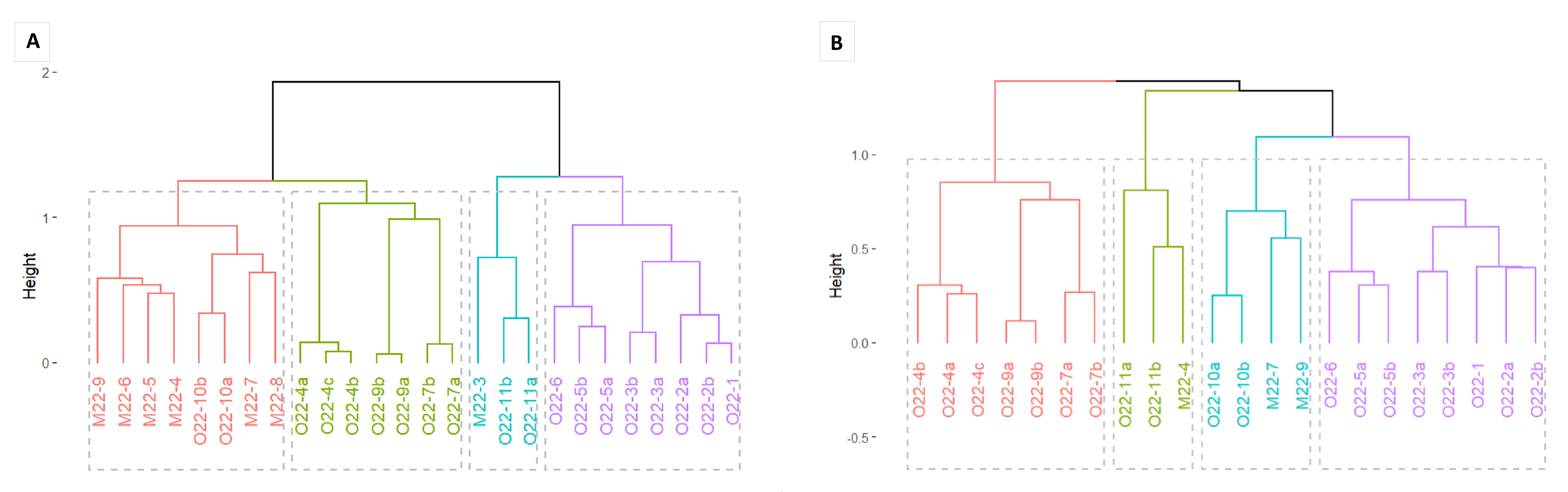

Supplement: Supplementary file 6 — FIGURE S6: Hierarchical agglomerative clustering based on UniFrac distance matrices and the Ward method for (A) bacterial and (B) archaeal community composition. Letters a, b, c following sample names denote replicates. Coloured clusters highlight four phylogenetically distinct groups that have been reported on the PCoA results (Figure 6). [file EMI4-17-e70238-s007.png]
