# Supplementary material for: Groundwater Microbial Diversity Associated With Icelandic Basaltic Subsurface Environments
Source: Environ Microbiol Rep. 2025 Nov 30;17(6):e70238. doi: 10.1111/1758-2229.70238 (PMC12665481; doi:10.1111/1758-2229.70238)
Supplement: Supplementary file 7 — TABLE S1: Characteristics and coordinates of the wells sampled (see Figure 1 for locations). Information on wells were retrieved from well‐specific reports deposited in the database operated by the Icelandic National Energy Authority (Orkustofnun; https://www.map.is/os/) and Jóhannesson (2014). Each DNA concentration value corresponds to the mean of three measurements. Where applicable, ranges are shown for wells with replicates. The ‘PCR amplification’ column indicates the outcome of the 16S rRNA gene amplification using domain‐specific primers: ‘+’ denotes successful amplification for both bacteria and archaea; ‘(−)’ indicates failure for archaea only; and ‘–’ means amplification failed for both domains. TABLE S2: Taxonomic classification and closest cultivated (‘Accession (cult.)’) and environmental (‘Accession (env.)’) relatives—along with the environments from which they were retrieved—of the 20 most abundant bacterial ASVs (ranked similarly to Figure 5) identified in groundwater samples and considered for community analysis using the FROGS pipeline (Escudié et al. 2018) (based on sequence similarity analyses using BLAST searches (Camacho et al. 2009) against the NCBI non‐redundant nucleotide database). Ca., Candidatus. TABLE S3: Taxonomic classification and closest cultivated (‘Accession (cult.)’) and environmental (‘Accession (env.)’) relatives—along with the environments from which they were retrieved—of the 20 most abundant archaeal ASVs (ranked similarly to Figure 5) identified in groundwater samples and considered for community analysis using the FROGS pipeline (Escudié et al. 2018) based on sequence similarity analyses using BLAST searches (Camacho et al. 2009) against the NCBI non‐redundant nucleotide database. Ca., Candidatus. [file EMI4-17-e70238-s002.docx]

**SUPPLEMENTARY TABLES**

**TABLE S1** Characteristics and coordinates of the wells sampled (see Figure 1 for locations). Information on wells were retrieved from well-specific reports deposited in the database operated by the Icelandic National Energy Authority (Orkustofnun; https://www.map.is/os/) and Jóhannesson (2014). Each DNA concentration value corresponds to the mean of three measurements. Where applicable, ranges are shown for wells with replicates. The ‘PCR amplification’ column indicates the outcome of the 16S rRNA gene amplification using domain-specific primers: ‘+’ denotes successful amplification for both bacteria and archaea; ‘(–)’ indicates failure for archaea only; and ‘–’ means amplification failed for both domains.

| **Well** | **Coordinates** | **Depth**^a^  (m) | **Bedrock age** (Myr) | **DNA concentration**  (ng μL^-1^) | **PCR amplification** |
| --- | --- | --- | --- | --- | --- |
| M22-1 | 64.162172°, -22.009550° | 2714 | 0.011 - 0.8 | <d.l.^b^ | - |
| M22-2 | 64.160484°, -22.005189° | 2207 | 0.011 - 0.8 | 0.025 | - |
| M22-3 | 64.118063°, -21.824944° | 1605 | 0.011 - 0.8 | <d.l. | (-) |
| M22-4 | 64.113073°, -21.829704° | 1615 | 0.011 - 0.8 | 0.068 | + |
| M22-5** | 64.152976°, -21.654014° | 1203 | 0.011 - 0.8 | 0.144 | + |
| M22-6 | 64.157004°, -21.650830° | 1604 | 0.8 - 3.3 | n.d.^c^ | (-) |
| M22-7 | 64.068953°, -20.644045° | 101 | 0.8 - 3.3 | <d.l. | + |
| M22-8** | 64.147294°, -20.553406° | 63 | 0.8 - 3.3 | 0.035 | + |
| M22-9 | 64.043895°, -20.448672° | 100 | 0.8 - 3.3 | 0.037 | + |
| M22-10 | 64.139222°, -21.893934° | 1360 | 0.011 - 0.8 | 0.092 | - |
| M22-11 | 63.917163°, -20.411034° | 1847 | 0.011 - 0.8 | <d.l. | - |
| O22-1 | 65.872572°, -17.452096° | 101 | 0.011 - 0.8 | 0.026 | + |
| O22-2 | 65.872110°, -17.452877° | unknown | 0.011 - 0.8 | 0.012 - 0.018 | + |
| O22-3 | 65.722378°, -17.354707° | 687 | 0.8 - 3.3 | 0.013 - 0.014 | + |
| O22-4* | 65.821759°, -17.908410° | 63 | 3.3 - 15 | <d.l. or 0.030 - 0.130^d^ | + |
| O22-5 | 65.733191°, -19.616450° | 667 | 7 - 10 | 0.013 - 0.200 | + |
| O22-6 | 65.732611°, -19.616358° | 578 | 7 - 10 | <d.l. | + |
| O22-7 | 65.467840°, -19.352256° | unknown | 3.3 - 15 | 0.340 - 0.630 | + |
| O22-8 | 65.556306°, -19.457699° | 427 | 3.3 - 15 | <d.l. | - |
| O22-9 | 64.537181°, -21.701175° | 20 | 3.3 - 15 | 1.120 - 1.300 | + |
| O22-10 | 64.492672°, -21.176456° | unknown | 0.8 - 3.3 | 0.190 - 0.340 | + |
| O22-11* | 64.413079°, -21.901570° | 657 | 3.3 - 15 | 0.029 - 0.054 | + |

^a^refers to the bottom of the borehole.

^b^below detection limit.

^c^not determined due to insufficient sample quantity.

^d^depending on replicate.

*wells excluded from the ANOVA analysis due to incomplete input data.

**archaeal ASVs data excluded from community structure analysis due to insufficient sequencing depth and rarefaction curves not reaching a plateau (Figure S1B).

**TABLE S2** Taxonomic classification and closest cultivated (‘Accession (cult.)’) and environmental (‘Accession (env.)’) relatives—along with the environments from which they were retrieved—of the 20 most abundant bacterial ASVs (ranked similarly to Figure 5) identified in groundwater samples and considered for community analysis using the FROGS pipeline (Escudié et al. 2018) (based on sequence similarity analyses using BLAST searches (Camacho et al. 2009) against the NCBI non-redundant nucleotide database). *Ca.*, *Candidatus*.

| **ASV ID** | **BLAST taxonomy** | **Accession (env.)** | **Identity** (%) | **Environment** | **Accession (cult.)** | **Identity** (%) | **Environment** | **References** |
| --- | --- | --- | --- | --- | --- | --- | --- | --- |
| Cluster_14 | Bacteria; Verrucomicrobiota; Verrucomicrobiae; Opitutales; Puniceicoccaceae; *Puniceicoccus*; unknown species | MG601424 | 80.06 | sinkhole, Bahamas, USA | none |  |  |  |
| Cluster_12 | Bacteria; Verrucomicrobiota; Verrucomicrobiae; Opitutales; Puniceicoccaceae; *Puniceicoccus*; unknown species | EU385871 | 81.32 | subseafloor sediment of the South China sea | none |  |  |  |
| Cluster_27 | Bacteria; Verrucomicrobiota; Verrucomicrobiae; Opitutales; Puniceicoccaceae; *Puniceicoccus*; unknown species | JN523467 | 80.98 | hypersaline evaporation pond, Guerrero Negro | none |  |  |  |
| Cluster_11 | Bacteria; Nitrospirota; Thermodesulfovibrionia; unknown | KC831434 | 95.71 | Betong hot spring, Yala province, Thailand | none |  |  |  |
|  | order; unknown family; unknown genus; unknown species | FJ206255 | 95.71 | alkaline thermal spring, Yellowstone, USA | none |  |  |  |
| Cluster_9 | Bacteria; Nitrospirota; Thermodesulfovibrionia; Thermodesulfovibrionales; Thermodesulfovibrionaceae; *Thermodesulfovibrio*; unknown species | FJ936959 | 94.92 | mud volcano, Kamchatka | none |  |  |  |
|  | Bacteria; Nitrospirota; Thermodesulfovibrionia; Thermodesulfovibrionales; Thermodesulfovibrionaceae; *Thermodesulfovibrio*; unidentified *Nitrospira* group OPB67A | AF027001 | 94.91 | Yellowstone hot spring, USA | none |  |  |  |
| Cluster_6 | Bacteria; Nitrospirota; Thermodesulfovibrionia; unknown | KX163922 | 99.20 | basaltic subsurface ecosystems, Iceland | none |  |  |  |
|  | order; unknown family; unknown genus; unknown species | KX163716 | 99.20 | basaltic subsurface ecosystems, Iceland | none |  |  |  |
| Cluster_18 | Bacteria; Nitrospirota; Thermodesulfovibrionia; unknown | KX163716 | 99.73 | basaltic subsurface ecosystems, Iceland | none |  |  |  |
|  | order; unknown family;unknown genus; unknown species | KX163922 | 99.74 | basaltic subsurface ecosystems, Iceland | none |  |  |  |
| Cluster_22 | Bacteria; Nitrospirota; Thermodesulfovibrionia; unknown | KX163301 | 99.20 | basaltic subsurface ecosystems, Iceland | none |  |  |  |
|  | order; unknown family; unknown genus; unknown species | KX163313 | 99.20 | basaltic subsurface ecosystems, Iceland | none |  |  |  |
|  |  | KX163302 | 99.20 | basaltic subsurface ecosystems, Iceland | none |  |  |  |
|  |  | KX163310 | 99.20 | basaltic subsurface ecosystems, Iceland | none |  |  |  |
|  |  | KX163550 | 99.20 | basaltic subsurface ecosystems, Iceland | none |  |  |  |
| Cluster_5 | Bacteria; Nitrospirota; Thermodesulfovibrionia; Thermodesulfovibrionales; Thermodesulfovibrionaceae; *Thermodesulfovibrio*; unidentified *Nitrospira* group OPB67A | AF027001 | 100 | Yellowstone hot spring, USA | none |  |  |  |
| Cluster_45 | Bacteria; Nitrospirota; Thermodesulfovibrionia; Thermodesulfovibrionales; Thermodesulfovibrionaceae; Thermodesulfovibrio; unknown species | FJ936959 | 95.19 | mud volcano, Kamchatka | none |  |  |  |
| Cluster_2 | Bacteria; Nitrospirota; Nitrospiria; Nitrospirales; Nitrospiraceae; *Nitrospira*; unknown species | KP204488 | 100 | Copahue geothermal field, Argentina | *Ca. Nitrospira inopinata* LN885086 | 100 | pipe under the flow of hot water (56°C) | Daims et al. (2015) |
|  |  | FJ936796 | 100 | mud volcano, Kamchatka | *Ca. Nitrospira inopinata* LN885086 | 100 | pipe under the flow of hot water (56°C) | Daims et al. (2015) |
|  |  | FR846897.1 | 100 | subsurface thermal spring | *Ca. Nitrospira inopinata* LN885086 | 100 | pipe under the flow of hot water (56°C) | Daims et al. (2015) |
|  | Bacteria; Nitrospirota; Nitrospiria; Nitrospirales; Nitrospiraceae; *Nitrospira*; *Ca. Nitrospira inopinata* | LN885086.1 | 100 | pipe under the flow of hot water (56°C) | *Ca. Nitrospira inopinata* LN885086 | 100 | pipe under the flow of hot water (56°C) | Daims et al. (2015) |
| Cluster_4 | Bacteria; Nitrospirota; Thermodesulfovibrionia; Thermodesulfovibrionales; Thermodesulfovibrionaceae; *Thermodesulfovibrio*; unknown species | HM595393 | 99.73 | mud from geothermal soil, Iceland | *Thermodesulfovibrio aggregans* NR_040795 | 95.71 | thermophilic methanogenic sludges | Sekiguchi et al. (2008 |
| Cluster_1 | Bacteria; Nitrospirota; Thermodesulfovibrionia; unknown | KX163922 | 98.93 | basaltic subsurface ecosystems, Iceland | none |  |  |  |
|  | order; unknown family; unknown genus; unknown species | KX163716 | 98.93 | basaltic subsurface ecosystems, Iceland | none |  |  |  |

| Cluster_73 | Bacteria; Firmicutes; Desulfotomaculia; Ammonifexales; Desulforudaceae; *Ca. Desulforudis*; *Ca. Desulforudis* | KF939343 | 100 | deep geothermal aquifer | *Ca. Desulforudis audaxviator* CP034260.2 | 100 | deep aquifer, Siberia | Karnachuk et al. (2019) |
| --- | --- | --- | --- | --- | --- | --- | --- | --- |
|  | *audaxviator* MP104C | EU730988 | 100 | fracture water, deep gold mine, South Africa | *Ca. Desulforudis audaxviator* CP034260.2 | 100 | deep aquifer, Siberia | Karnachuk et al. (2019) |
|  |  | KX163967 | 100 | basaltic subsurface ecosystems, Iceland | *Ca. Desulforudis audaxviator* CP034260.2 | 100 | deep aquifer, Siberia | Karnachuk et al. (2019) |
|  |  | DQ088810 | 100 | deep crustal groundwater, South Africa | *Ca. Desulforudis audaxviator* CP034260.2 | 100 | deep aquifer, Siberia | Karnachuk et al. (2019) |
|  |  | EU730980 | 100 | fracture water, deep gold mine, South Africa | *Ca. Desulforudis audaxviator* CP034260.2 | 100 | deep aquifer, Siberia | Karnachuk et al. (2019) |
| Cluster_15 | Bacteria; Desulfobacterota; Thermodesulfobacteria; Thermodesulfobacteriales; Thermodesulfobacteriaceae; *Geothermobacterium*; *Thermodesulfotobacterium* sp. | AF255595 | 100 | hot spring microbial mats, Iceland | *Caldimicrobium rimae* NR_044283 | 95.97 | Uzon caldera, Kamchatka, Russia | Miroshnichenko et al. (2009) |
| Cluster_16 | Bacteria; Desulfobacterota; Syntrophorhabdia; Syntrophorhabdales; Syntrophorhabdaceae; *Syntrophorhabdus*; unknown species | AB704739 | 85.94 | 250 m depth borehole, Hokkaido, Japan | none |  |  |  |
| Cluster_7 | Bacteria; Desulfobacterota; unknown class; unknown order; | AF026994 | 99.47 | Yellowstone hot spring, USA | none |  |  |  |
|  | unknown family; unknown genus; unknown species | GQ844347 | 99.47 | biofilm from an anoxic tank, China | none |  |  |  |
|  |  | AF026993 | 99.20 | Yellowstone hot spring, USA | none |  |  |  |
|  |  | AY861742 | 99.20 | Yellowstone geothermal ecosystem, USA | none |  |  |  |
|  |  | KX213922 | 98.94 | Yellowstone hot spring, USA | none |  |  |  |
| Cluster_3 | Bacteria; Deinococcota; Deinococci; Thermales; Thermaceae; | HQ750124.1 | 100 | gastrointestinal specimens | *Allomeiothermus silvanus* Y13598 | 100 | hot springs within Geysir hydrothermal field, Iceland | Chung et al. (1997) |
|  | *Meiothermus*; unknown species | HQ742914.1 | 100 | gastrointestinal specimens | *Allomeiothermus silvanus* Y13598 | 100 | hot springs within Geysir hydrothermal field, Iceland | Chung et al. (1997) |
|  |  | HQ749629.1 | 100 | gastrointestinal specimens | *Allomeiothermus silvanus* Y13598 | 100 | hot springs within Geysir hydrothermal field, Iceland | Chung et al. (1997) |
|  |  | HQ750050.1 | 100 | gastrointestinal specimens | *Allomeiothermus silvanus* Y13598 | 100 | hot springs within Geysir hydrothermal field, Iceland | Chung et al. (1997) |
|  |  | HQ750815.1 | 100 | gastrointestinal specimens | *Allomeiothermus silvanus* Y13598 | 100 | hot springs within Geysir hydrothermal field, Iceland | Chung et al. (1997) |
| Cluster_8 | Bacteria; Aquificota; Aquificae; Aquificales; Aquificaceae; | AM259501.1 | 100 | Geyser Valley, Kamchatka, Russia | *Thermothrix azorensis* NR_104832 | 99.73 | São Miguel Island hot spring, Azores | Odintsova et al. (1996) |
|  | *Hydrogenobacter*; unknown species | LC375847.1 | 100 | Nakabusa hot spring, Japan | *Thermothrix azorensis* NR_104832 | 99.73 | São Miguel Island hot spring, Azores | Odintsova et al. (1996) |
|  |  | KP175576.1 | 100 | Rehai geothermal field, Yunnan, China | *Thermothrix azorensis* NR_104832 | 99.73 | São Miguel Island hot spring, Azores | Odintsova et al. (1996) |
|  |  | EU815128.1 | 100 | thermal vent boiling pool, 850, Tibet, China | *Thermothrix azorensis* NR_104832 | 99.73 | São Miguel Island hot spring, Azores | Odintsova et al. (1996) |
|  |  | EU815160.1 | 100 | thermal vent boiling pool, 850, Tibet, China | *Thermothrix azorensis* NR_104832 | 99.73 | São Miguel Island hot spring, Azores | Odintsova et al. (1996) |
| Cluster_38 | Bacteria; Aquificota; Aquificae; Aquificales; Aquificaceae; *Thermocrinis*; unknown species | AF255599 | 100 | hot spring microbial mats, Iceland | *Thermocrinis albus* CP001931, NR_075037 | 100 | whitish steamers of the Hveragerthi area, Iceland | Wirth et al. (2010) |
|  |  | GU233812 | 100 | Icelandic geothermal waters | *Thermocrinis albus* CP001931, NR_075037 | 100 | whitish steamers of the Hveragerthi area, Iceland | Wirth et al. (2010) |
|  |  | HQ899614 | 100 | volcanic ash at Eyjafjallajokull, Iceland | *Thermocrinis albus* CP001931, NR_075037 | 100 | whitish steamers of the Hveragerthi area, Iceland | Wirth et al. (2010) |
|  | Bacteria; Aquificota; Aquificae; Aquificales; Aquificaceae; *Thermocrinis*; *Thermocrinis albus* | CP001931 | 100 | whitish steamers of the Hveragerthi area, Iceland | *Thermocrinis albus* CP001931, NR_075037 | 100 | whitish steamers of the Hveragerthi area, Iceland | Wirth et al. (2010) |
|  |  | NR_075037 | 100 | whitish steamers of the Hveragerthi area, Iceland | *Thermocrinis albus* CP001931, NR_075037 | 100 | whitish steamers of the Hveragerthi area, Iceland | Wirth et al. (2010 |

**TABLE S3** Taxonomic classification and closest cultivated (‘Accession (cult.)’) and environmental (‘Accession (env.)’) relatives—along with the environments from which they were retrieved—of the 20 most abundant archaeal ASVs (ranked similarly to Figure 5) identified in groundwater samples and considered for community analysis using the FROGS pipeline (Escudié et al. 2018 (based on sequence similarity analyses using BLAST searches (Camacho et al. 2009) against the NCBI non-redundant nucleotide database). *Ca.*, *Candidatus*.

| **ASV ID** | **BLAST taxonomy** | **Accession (env.)** | **Identity** (%) | **Environment** | **Accession (cult.)** | **Identity** (%) | **Environment** | **References** |
| --- | --- | --- | --- | --- | --- | --- | --- | --- |
| Cluster_19 | Archaea; Hadarchaeota; Hadarchaeia; unknown order; | DQ088781 | 97.09 | deep crustal groundwater, South Africa | none |  |  |  |
|  | Hadarchaeales; unknown genus; unknown species | DQ256304 | 97.09 | subsurface water of the Kalahari Shield, South Africa | none |  |  |  |
|  |  | DQ230938 | 97.09 | subsurface water of the Kalahari Shield, South Africa | none |  |  |  |
| Cluster_29 | Archaea; Hadarchaeota; Hadarchaeia; unknown order; Hadarchaeales; unknown genus; unknown species | NR690969 | 99.47 | alkaline groundwater, Portugal | none |  |  |  |
| Cluster_15 | Archaea; Hadarchaeota; Hadarchaeia; unknown order; | DQ088781 | 96.30 | deep crustal groundwater, South Africa | none |  |  |  |
|  | Hadarchaeales; unknown genus; unknown species | DQ256304 | 96.30 | subsurface water of the Kalahari Shield, South Africa | none |  |  |  |
|  |  | DQ256308 | 96.30 | subsurface water of the Kalahari Shield, South Africa | none |  |  |  |
|  |  | DQ230938 | 96.30 | subsurface water of the Kalahari Shield, South Africa | none |  |  |  |
| Cluster_26 | Archaea; Hadarchaeota; Hadarchaeia; unknown order; Hadarchaeales; unknown genus; unknown species | NR690975 | 94.44 | alkaline groundwater, Portugal | none |  |  |  |
| Cluster_18 | Archaea; Hadarchaeota; Hadarchaeia; unknown order; | AB050226 | 96.03 | deep gold mine water, South Africa | none |  |  |  |
|  | Hadarchaeales; unknown genus; unknown species | AB050212 | 96.03 | deep gold mine water, South Africa | none |  |  |  |
| Cluster_43 | Archaea; Hadarchaeota; Hadarchaeia; unknown order; Hadarchaeales; unknown genus; unknown species | KJ149161 | 99.47 | carbonate chimney, Prony Hydrothermal Field, New Caledonia | none |  |  |  |
|  |  | KJ149162 | 99.47 | carbonate chimney, Prony Hydrothermal Field, New Caledonia | none |  |  |  |
| Cluster_1 | Archaea; Crenarchaeota; Nitrososphaeria; Nitrosocaldales; Nitrosocaldaceae; *Ca. Nitrosocaldus*; unknown species | HM448128 | 98.94 | streamer biofilm community, Yellowstone, USA | *Ca. Nitrosocaldus yellowstonensis* EU239960 | 98.67 | hot spring Yellowstone, USA | De La Torre et al. (2008) |
|  |  | HQ395707 | 98.94 | hot spring, Kamchatka, Russia | *Ca. Nitrosocaldus yellowstonensis* EU239960 | 98.67 | hot spring Yellowstone, USA | De La Torre et al. (2008) |
|  |  | LN794726 | 98.67 | well water of a geothermal karst system, Budapest, Hungary | *Ca. Nitrosocaldus yellowstonensis* EU239960 | 98.67 | hot spring Yellowstone, USA | De La Torre et al. (2008) |
|  |  | HQ395706 | 98.67 | hot spring, Kamchatka, Russia | *Ca. Nitrosocaldus yellowstonensis* EU239960 | 98.67 | hot spring Yellowstone, USA | De La Torre et al. (2008) |
|  |  | HM448103 | 98.67 | streamer biofilm community, Yellowstone, USA | *Ca. Nitrosocaldus yellowstonensis* EU239960 | 98.67 | hot spring Yellowstone, USA | De La Torre et al. (2008) |
| Cluster_2 | Archaea; Crenarchaeota; Nitrososphaeria; | JF262288 | 100 | Yellowstone lake, USA | *Ca. Nitrosotenuis aquarius* KX023321 | 98.94 | fresh water aquarium biofilter, Canada | (Sauder et al. 2018 |
|  | Nitrosopumilales; Nitrosopumilaceae; *Ca. Nitrosotenuis*; | JF262322 | 100 | Yellowstone lake, USA | *Ca. Nitrosotenuis aquarius* KX023321 | 98.94 | fresh water aquarium biofilter, Canada | (Sauder et al. 2018 |
|  | unknown species | JF262296 | 100 | Yellowstone lake, USA | *Ca. Nitrosotenuis aquarius* KX023321 | 98.94 | fresh water aquarium biofilter, Canada | (Sauder et al. 2018 |
|  |  | JF262289 | 100 | Yellowstone lake, USA | *Ca. Nitrosotenuis aquarius* KX023321 | 98.94 | fresh water aquarium biofilter, Canada | (Sauder et al. 2018 |
|  |  | JF262284 | 100 | Yellowstone lake, USA | *Ca. Nitrosotenuis aquarius* KX023321 | 98.94 | fresh water aquarium biofilter, Canada | (Sauder et al. 2018 |
| Cluster_3 | Archaea; Crenarchaeota; Methanomethylicia; Methanomethyliales; Methanomethyliaceae; | KP784721 | 100 | hot spring at Tengchong geothermal field, China | *Ca. Methanosuratincola petrocarbonis* CP130488 | 97.61 | oil field reservoir, China | Wu et al. (2025) |
|  | *Ca. Methanomethylicus*; unknown species | KM221243 | 100 | hot spring at Tengchong geothermal field, China | *Ca. Methanosuratincola petrocarbonis* CP130488 | 97.61 | oil field reservoir, China | Wu et al. (2025) |
|  |  | KM585418 | 100 | thermal soil at Tengchong geothermal field, China | *Ca. Methanosuratincola petrocarbonis* CP130488 | 97.61 | oil field reservoir, China | Wu et al. (2025) |
|  |  | AY861923 | 100 | Yellowstone geothermal ecosystem, USA | *Ca. Methanosuratincola petrocarbonis* CP130488 | 97.61 | oil field reservoir, China | Wu et al. (2025) |
|  |  | AY861911 | 100 | Yellowstone geothermal ecosystem, USA | *Ca. Methanosuratincola petrocarbonis* CP130488 | 97.61 | oil field reservoir, China | Wu et al. (2025) |

| Cluster_5 | Archaea; Crenarchaeota; Nitrososphaeria; Nitrosocaldales; Nitrosocaldaceae; *Ca. Nitrosocaldus*; unknown species | AF361211 | 99.73 | subterranean hot spring, Iceland | *Ca. Nitrosocaldus yellowstonensis* EU239960 | 98.94 | hot spring Yellowstone, USA | De La Torre et al. (2008) |
| --- | --- | --- | --- | --- | --- | --- | --- | --- |
|  |  | HQ395707 | 99.73 | hot spring, Kamchatka, Russia | *Ca. Nitrosocaldus yellowstonensis* EU239960 | 98.94 | hot spring Yellowstone, USA | De La Torre et al. (2008) |
|  |  | LN794726 | 99.47 | well water of a geothermal karst system, Budapest, Hungary | *Ca. Nitrosocaldus yellowstonensis* EU239960 | 98.94 | hot spring Yellowstone, USA | De La Torre et al. (2008) |
|  |  | HQ395706 | 99.47 | hot spring, Kamchatka, Russia | *Ca. Nitrosocaldus yellowstonensis EU239960* | 98.94 | hot spring Yellowstone, USA | De La Torre et al. (2008) |
|  |  | LN794707 | 99.47 | well water of a geothermal karst system, Budapest, Hungary | *Ca. Nitrosocaldus yellowstonensis* EU239960 | 98.94 | hot spring Yellowstone, USA | De La Torre et al. (2008) |
| Cluster_4 | Archaea; Crenarchaeota; Nitrososphaeria; unknown order; unknown family; unknown genus; unknown species | KC437231 | 99.47 | spring water, Ash Meadows National Wildlife Refuge, Nevada, USA | none |  |  |  |
|  |  | KC437291 | 99.47 | spring water, Ash Meadows National Wildlife Refuge, Nevada, USA | none |  |  |  |
|  |  | KC437261 | 99.47 | spring water, Ash Meadows National Wildlife Refuge, Nevada, USA | none |  |  |  |
|  |  | KC437192 | 99.47 | spring water, Ash Meadows National Wildlife Refuge, Nevada, USA | none |  |  |  |
| Cluster_6 | Archaea; Crenarchaeota; Bathyarchaeia; unknown order; unknown family; unknown genus; unknown species | EU924240 | 97.35 | hot spring sediment from Little Hot Creek, California, USA | none |  |  |  |
|  |  | EU924225 | 97.35 | hot spring sediment from Little Hot Creek, California, USA | none |  |  |  |
|  |  | AY861927 | 97.35 | Yellowstone geothermal ecosystem, USA | none |  |  |  |
|  |  | EU635904 | 97.35 | hot spring sediment, Nevada, USA | none |  |  |  |
|  |  | EU635929 | 97.35 | hot spring sediment, Nevada, USA | none |  |  |  |
| Cluster_36 | Archaea; Crenarchaeota; Bathyarchaeia; unknown order; unknown family; unknown genus; unknown species | FR727664 | 94.29 | hot spring in the Aquitaine bassin, France | none |  |  |  |
| Cluster_7 | Archaea; Crenarchaeota; Thermoprotei; Sulfolobales; Sulfolobaceae; *Saccharolobus*; *Saccharolobus solfataricus* | CP033241 | 100 | volcanic hot spring, Campi Flegrei, Italy | *Saccharolobus solfataricus* CP033241, NR_119198 / *Sulfolobus islandicus* AY247897 | 100 | Brock et al. (1972), Whitaker et al. (2003), Payne et al. (2018) |  |
|  | Archaea; Crenarchaeota; Thermoprotei; Sulfolobales; Sulfolobaceae; *Saccharolobus*; *Sulfolobus* sp. G4ST-T-11 | FJ870913 | 100 | hot spring, Iceland | same as above | 100 | same as above |  |
|  | Archaea; Crenarchaeota; Thermoprotei; Sulfolobales; Sulfolobaceae; *Saccharolobus*; *Saccharolobus solfataricus* | NR_119198 | 100 | volcanic hot spring, Campi Flegrei, Italy | same as above | 100 | same as above |  |
|  | Archaea; Crenarchaeota; Thermoprotei; Sulfolobales; Sulfolobaceae; *Saccharolobus*; *Sulfolobus* sp. FF5/00 | AF425656 | 100 | hot spring, Yellowstone, USA | same as above | 100 | same as above |  |
|  | Archaea; Crenarchaeota; Thermoprotei; Sulfolobales; Sulfolobaceae; *Saccharolobus*; *Sulfolobus islandicus* | AY247897 | 100 | acidic hot springs, Iceland, USA, Russia | same as above | 100 | same as above |  |
| Cluster_8 | Archaea; Crenarchaeota; Thermoprotei; Desulfurococcales; Ignisphaeraceae; unknown genus; unknown species | FR852930 | 89.25 | active hydrothermal chimney, Juan de Fuca ridge | none |  |  |  |
| Cluster_22 | Archaea; Crenarchaeota; Nitrososphaeria; Caldiarchaeales; Caldiarchaeaceae; *Ca. Caldiarchaeum*; unknown species | KM221273 | 94.43 | hot spring at Tengchong geothermal field, China | none |  |  |  |

| Cluster_11 | Archaea; Crenarchaeota; Nitrososphaeria; Caldiarchaeales; Caldiarchaeaceae; *Ca. Caldiarchaeum*; unknown species | AY555827 | 99.47 | Bor Khlueng hot spring, Thailand | none |  |  |  |
| --- | --- | --- | --- | --- | --- | --- | --- | --- |
| Cluster_14 | Archaea; Crenarchaeota; Bathyarchaeia; unknown order; | KX213924 | 98.67 | hot spring, Yellowstone, USA | none |  |  |  |
|  | unknown family; unknown genus; unknown species | KM585431 | 98.14 | thermal soil at Tengchong geothermal field, China | none |  |  |  |
| Cluster_24 | Archaea; Crenarchaeota; Bathyarchaeia; unknown order; unknown family; unknown genus; unknown species | EU924227 | 93.72 | hot spring sediment from Little Hot Creek, California, USA | none |  |  |  |
| Cluster_33 | Archaea; Crenarchaeota; Nitrososphaeria; Caldiarchaeales; | KP091637 | 99.47 | hot spring, Yellowstone, USA | none |  |  |  |
|  | Caldiarchaeaceae; *Ca. Caldiarchaeum*; | KT028690 | 99.47 | hot spring sediment, Nevada, USA | none |  |  |  |
|  | Thaumarchaeota archaeon JGI OTU-1 | EU635911 | 99.47 | hot spring sediment, Nevada, USA | none |  |  |  |
|  |  | EF100632 | 99.47 | submarine hydrothermal system, Tutum Bay, Papua New Guinea | none |  |  |  |

**SUPPLEMENTARY FIGURES**

**FIGURE S1** Rarefaction curves for (**A**) bacterial and (**B**) archaeal communities across all groundwater samples. The dashed green and blue lines indicate richness values at 18,038 and 6,286 sequences, the thresholds used to rarefy the bacterial and archaeal ASV tables, respectively (see Methods). For clarity, the *x*-axis (*i.e.*, sample size) was truncated at 50,000 sequences for bacteria and 20,000 sequences for archaea.

**FIGURE S2** Observed number of ASVs as a function of wellhead temperature (°C) for each groundwater sample in which DNA extraction and PCR amplification of the 16S rRNA gene were successful. Results are shown separately for (**A**) bacterial and (**B**) archaeal communities.

**FIGURE S3** Relative abundance of the most dominant microbial phyla retrieved from groundwater samples, shown separately for (**A**) bacteria (10 most abundant phyla) and (**B**) archaea (6 most abundant phyla). Taxonomic assignments were generated using the FROGS pipeline (Escudié et al. 2018) based on the SILVA rRNA gene database (release 138.1; Quast et al. 2012). For each well, the measured wellhead temperature and pH at ~20°C (pH_20°C_) are indicated above the corresponding bar. Samples are ordered by increasing temperature (from 30 to 93°C for bacteria and from 30 to 87°C for archaea). Letters (a, b, c) following sample names denote replicates.

**FIGURE S4** Relative abundance of the most dominant microbial genera retrieved from groundwater samples, shown separately for (**A**) bacteria (30 most abundant genera) and (**B**) archaea (20 most abundant genera). Taxonomic assignments were generated using the FROGS pipeline (Escudié et al. 2018) based on the SILVA rRNA gene database (release 138.1; Quast et al. 2012). For each well, the measured wellhead temperature and pH at ~20°C (pH_20°C_) are indicated above the corresponding bar. Samples are ordered by increasing temperature (from 30 to 93°C for bacteria and from 30 to 87°C for archaea). Letters (a, b, c) following sample names denote replicates. See also Tables S2 and S3 for sequence similarity analyses using the BLAST+ tool (Camacho et al. 2009).

**FIGURE S5** Relative abundance of the most dominant microbial species retrieved from groundwater samples, shown separately for (**A**) bacteria (30 most abundant species) and (**B**) archaea (20 most abundant species. These 20 species represent the only confidently identified taxa across the dataset). Taxonomic assignments were generated using the FROGS pipeline (Escudié et al. 2018) based on the SILVA rRNA gene database (release 138.1; Quast et al. 2012). For each well, the measured wellhead temperature and pH at ~20°C (pH_20°C_) are indicated above the corresponding bar. Samples are ordered by increasing temperature (from 30 to 93°C for bacteria and from 30 to 87°C for archaea). Letters (a, b, c) following sample names denote replicates.See also Tables S2 and S3 for sequence similarity analyses using the BLAST+ tool (Camacho et al. 2009).

**FIGURE S6** Hierarchical agglomerative clustering based on UniFrac distance matrices and the Ward method for (**A**) bacterial and (**B**) archaeal community composition. Letters a, b, c following sample names denote replicates. Colored clusters highlight four phylogenetically distinct groups that have been reported on the PCoA results (Figure 6).
